# Electroactive Co(iii) salen metal complexes and the electrophoretic deposition of their porous organic polymers onto glassy carbon†

**DOI:** 10.1039/c8ra04385j

**Published:** 2018-07-03

**Authors:** Marcello B. Solomon, Aditya Rawal, James M. Hook, Seth M. Cohen, Clifford P. Kubiak, Katrina A. Jolliffe, Deanna M. D'Alessandro

**Affiliations:** School of Chemistry, The University of Sydney New South Wales 2006 Australia deanna.dalessandro@sydney.edu.au kate.jolliffe@sydney.edu.au +61 2 9351 3329 +61 2 9351 3777; NMR Facility, Mark Wainwright Analytical Centre, The University of New South Wales 2052 Australia; Department of Chemistry and Biochemistry, University of California San Diego California 92093 USA

## Abstract

This paper reports the CO_2_ electroreduction properties of three bis-bromo Co(iii) salen metal complexes and their Porous Organic Polymers (POPs) as a platform for using the salen core as a multi-electron reducing agent. Although Co(iii) salen metal complexes have been studied extensively for their chemical catalysis with CO_2_, their electrochemical behaviour, particularly their reduction, in the presence of CO_2_ is much less explored. The discrete Co(iii) complexes enabled the reduction of CO_2_ to CO in faradaic efficiencies of up to 20%. The reductive electrochemical processes of Co(iii) salen complexes are relatively unknown; therefore, the mechanism of reduction for the complexes was investigated using IR and UV-Vis-NIR spectroelectrochemical (SEC) techniques. The discrete bis-bromo salen complexes were incorporated into POPs with tris-(*p*-ethynyl)-triphenylamine as a co-ligand and were characterised using solid state NMR, IR, UV-Vis-NIR and Field Emission Scanning Electron Microscopy (FE-SEM). The POP materials were electrophoretically deposited onto glassy carbon under milder conditions than those previously reported in the literature. Direct attachment of the POP materials to glassy carbon enabled improved solid state electrochemical analysis of the samples. The POP materials were also analysed *via* SEC techniques, where a Co(ii/i) process could be observed, but further reductions associated with the imine reduction compromised the stability of the POPs.

## Abstract 2

We report the synthesis, characterisation and electrochemistry of Co(iii) salen metal complexes and their porous organic polymers for CO_2_ electroreduction.

## Graphical abstract

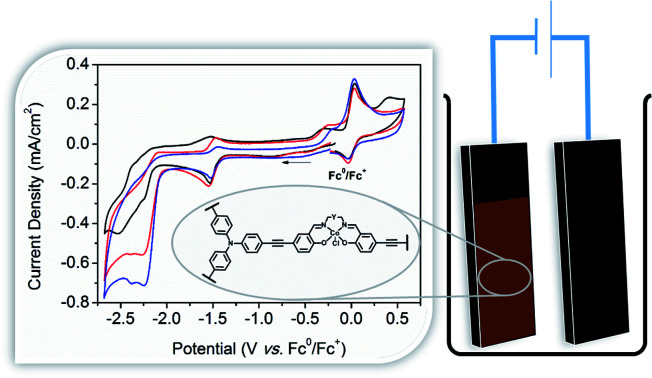

## Introduction

Since the industrial revolution, the consumption of fossil fuels has been rapidly increasing to cater for the needs of an ever growing world population. Industrialisation has been heavily dependent on the use of coal, which today is responsible for over 40% of the production of electricity worldwide.^1^ In excess of 12 million tonnes of oil and 8 million cubic tonnes of natural gas are consumed daily to provide energy, which has resulted in excess carbon dioxide (CO_2_) emissions into the atmosphere at a rate faster than the current carbon cycle can mitigate.^2–10^ Carbon capture and sequestration (CCS) has long been seen as a viable option as it allows for the retrofitting of existing power plants to separate CO_2_ from the flue stream prior to its release into the atmosphere.^11^ A concern arising from the storage of CO_2_ is its effective transport, which poses financial, logistical and environmental challenges. Other chief concerns for CCS are the safety and environmental aspects that arise from the high pressures and large concentrations of CO_2_. Processes that can combine the capture of CO_2_ with its use as a feedstock may assist in the handling of emissions.

The benefit of using CO_2_ as a feedstock lies in its abundance and low cost; however, its use as a cheap feedstock is limited by the presence of C in its most oxidised form. Investing energy to convert CO_2_ into a more reactive intermediate could enable the generation of commodity chemicals, which could eventually provide a cleaner fuel source for the human population to use. From a thermodynamic viewpoint, CO_2_ can accept an electron to form a CO_2_˙^−^ radical anion, but this is only observed directly at high reduction potentials (*E*_pc_ = −1.90 V *vs.* NHE).^12,13^ The proton-coupled reduction of CO_2_ provides a feasible alternative to direct reduction and can lead to the generation of useful products (carbon monoxide or formic acid) at lower reduction potentials.^13,14^ It is important that there is a kinetic preference for CO_2_ reduction over H_2_ due to the required presence of a proton source to facilitate the process.

Porous Organic Polymers (POPs) are considered viable candidates as materials that can both capture and convert CO_2_ into commodity chemicals. They are generally cheap to produce, simple to synthesise on a larger scale, possess high thermal and chemical stability,^15^ are lightweight, contain hydrophobic pores from their extended aromaticity and are chemically resistant. Hupp and co-workers have previously shown that the porosity and stability of POPs can be exploited by generating a polymer based on cheap and abundant amine- and anhydride-bearing monomers.^16^ The activated polymer demonstrated stability up to 500 °C and retained porosity after exposure to HCl. Patel and co-workers explored N_2_-phobicity within POPs by generating a class of nitrogen-rich azo POPs, which possessed high point selectivity for CO_2_/N_2_ (*S* = 288 at 323 K) and were chemically stable in boiling water.^17^ Zhou and co-workers synthesised POPs containing tetratopic adamantane cages which exhibited BET surface areas up to 5400 m^2^ g^−1^ and were suitable for CO_2_ and CH_4_ separation.^18^ It was found through ideal adsorbed solution theory (IAST) that several high surface area POPs demonstrated selectivity for CO_2_ at 295 K, suggesting their suitability for CO_2_ capture. Many early studies of POPs exploited porosity as their most important property; however, subsequent studies identify that these materials can also have applications in catalysis.^19^ Bare metal sites can also be incorporated into POPs, which may promote their use in catalysis.^20,21^

**Fig. 1:**
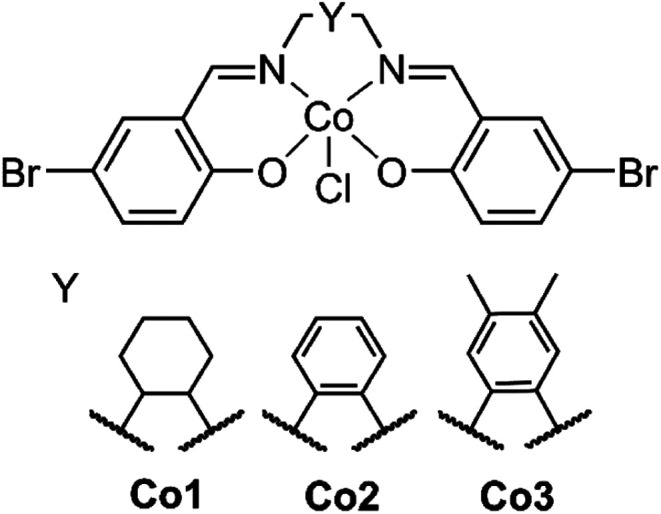
The structure of the salen metal complexes used here. The backbone can be systematically modified by varying Y to produce the analogs Co1, Co2 and Co3.

Creating a class of solid state materials that is capable of both the capture of CO_2_ and its catalytic conversion requires the choice of multifunctional building blocks. The ubiquity of salen metal complexes over the past eighty years has shown that they are capable of diverse chemistry. Since the first reported preparation of the vibrantly coloured salen-based ligands in 1933 by Pfeiffer,^22^ this class of ligand has found extensive use in chemical catalysts,^23^ electrochemical agents,^24^ and charge transfer complexes,^25^ among other applications. The variability of substituents on the salen core enables the preparation of diverse libraries of compounds that may be generated by systematic variation.^26^ There is scope to modify substituents along the backbone of the salen ligand to study their steric and electronic influence on catalysis (Fig. 1). The inherently redox-active nature of the salen metal complex allows for its properties to be modulated.

The readily functionalisable core of the salen metal complex allows for its covalent immobilisation into a POP material. A class of salen-based POPs were reported by Chun and co-workers, who varied the bridging moiety in the salen to incorporate the diaminocyclohexane functionality, and introduced Al(iii), Co(iii) and Cr(iii) metals into the chelating site. Not only did these polymers exhibit similar BET surface areas of 522–650 m^2^ g^−1^, but they also converted CO_2_ to cyclic carbonates with yields of up to 94%.^20^ Xie and co-workers also used POPs to examine the capture of CO_2_ and its conversion to propylene carbonate under mild conditions.^27^ Surface areas up to 965 m^2^ g^−1^ were achieved and the catalyst could be recycled up to 22 times without any significant decrease in activity. To the best of our knowledge, work has not yet appeared on the electrochemical reduction of salen-based POPs.

The electrochemical examination of POP materials requires their effective attachment to a conductive surface.^28^ A large proportion of the literature addressing the surface attachment of solid state materials (in particular Metal Organic Frameworks (MOFs)) onto a conductive surface has reported Indium-doped Tin Oxide (ITO) or Fluorine-doped Tin Oxide (FTO) on glass as the substrate of choice.^29–33^ These two substrates are both transparent, and therefore allow for the spectroscopic study of the surface attached material prior to, during and after reduction experiments using transmission measurements;^34^ however, there are limitations to their use. For example, ITO readily corrodes from glass substrates upon the application of highly anodic and cathodic potentials, and indium is a scarce and expensive element.^35^ Although FTO coated glass has a larger electrochemical window and uses more abundant fluorine, it has a high resistance, which affects the reversibility of the ferrocene/ferrocenium ion (Fc^0^/Fc^+^) couple.^36^ Glassy carbon is more conductive than FTO and ITO coated glass, and is therefore is a strong candidate as the substrate for solid state electrochemistry. The major limitation for glassy carbon is that mechanical immobilisation of materials such as POPs is difficult. Electrophoretic deposition (EPD) has been successfully applied to synthesise homogeneous, continuous films of UiO, MIL, NU,^30^ ZIF^37^ and porphyrin-based^38^ MOFs. The limitations of this particular surface attachment method lie in the high voltages required for deposition to occur. It is likely that immobilising of a POP onto glassy carbon using EPD could be achieved at lower voltages.^39^

Herein, we report the synthesis of three Co(iii) salen metal complexes to explore their use as electrochemical reducing agents for CO_2_. The electrochemical properties of the discrete complexes are analysed to examine the effect of modulating the bridging diamine functionality of the complexes on their electrochemical properties. We perform a detailed electrochemical study on the conversion of CO_2_ using these complexes to address the gap in the literature regarding salen complexes as reducing agents. We also report the inclusion of the discrete complexes into novel POPs and examine the impact of salen immobilisation on their redox properties. We further report a method for the surface attachment of POPs onto glassy carbon *via* EPD which uses milder potentials than those previously reported for EPD onto an FTO substrate, thus providing a benign pathway for attaching solid-state materials to glassy carbon.

## Experimental Methods

### General

All chemicals used were purchased from Aldrich, Alfa Aesar and Merck, and were used without further purification unless stated otherwise. Solvents were obtained from a PureSolv system, or purchased and used without further purification. Low resolution electrospray ionisation mass spectra (ESI-MS) were acquired as a solution in MeCN, MeOH or DMF with a 100 μL min^−1^ flow rate on a Finnegan LCQ or amazon MS detector. Spectra were collected over the mass range *m*/*z* 100 to 1000. An ESI spray voltage of 5 kV was applied with a heated capillary temperature of 200 °C and a nitrogen sheath gas pressure of 60 psi. Melting points were measured using a Gallenkamp melting point apparatus with the sample placed in a glass capillary. The final melting points are uncorrected. Room temperature FT-IR spectra were obtained using a PerkinElmer UATR 2 infrared spectrometer over the range 400–4000 cm^−1^ with a resolution of 4 cm^−1^. Samples were mechanically compressed on the surface of a diamond crystal on which the background was collected. ICP-OES was performed at the Mark Wainwright Analytical Centre at The University of New South Wales.

### Nuclear magnetic resonance

Solution state ^1^H and ^13^C{^1^H} NMR spectra were recorded on a Bruker AVANCEIII 300, 400 or 500 spectrometer operating at 300, 400, 500 MHz for ^1^H and 75, 100, 125 MHz for ^13^C, respectively. ^1^H and ^13^C NMR chemical shifts were referenced internally to residual solvent resonances. Spectra were recorded at 298 K and chemical shifts (*δ*), with uncertainties of ±0.01 Hz for ^1^H and ±0.05 Hz for ^13^C, are quoted in ppm. Coupling constants (*J*) are quoted in Hz and have uncertainties of ±0.05 Hz for ^1^H–^1^H. Deuterated solvents were obtained from Cambridge Stable Isotopes and used as received. Chemical shifts for all ligands are reported relative to the deuterated solvents used.^40^

The ^13^C cross polarisation magic angle spinning (CPMAS) solid state NMR experiments on the diamagnetic discrete complexes and their polymers were carried out at The Mark Wainwright Analytical Centre, The University of New South Wales, on a wide-bore Bruker Biospin AVANCEIII solids-300 MHz spectrometer operating at a frequency of 75 MHz for the ^13^C nucleus. The sample (∼80 mg) was placed into 4 mm zirconia rotors fitted with Kel-f® caps and spun in a double resonance H-X probehead at 8 kHz magic angle spinning (MAS). The ^13^C and ^1^H 90° radio frequency pulse lengths were optimised to 3.5 μs each. The ^13^C spectra were acquired with 1 ms cross polarisation contact time with a total suppression of spinning side bands (TOSS) scheme, followed by ^1^H decoupling at 75 kHz field strength using spinal-64 decoupling. The ^13^C non-quaternary suppression (NQS) spectra were recorded by turning off the ^1^H decoupling for 40 μs during the TOSS period. For sufficient signal-to-noise, *ca.* 10 K transients were acquired for each sample with recycle delays of 3.0 s in between to ensure sufficient relaxation of the ^1^H nuclei. The spectra were obtained at room temperature. The ^13^C chemical shifts were referenced to the glycine CO peak at 176 ppm.

### Cyclic voltammetry

Solution and solid state electrochemical measurements were performed using a Bioanalytical Systems BASi Epsilon Electrochemical Analyser at 298 K. A single compartment cell was used, consisting of either a glassy carbon working electrode (3.0 mm diameter) for solution state or a glassy carbon plate (active area of 2 cm^2^) with immobilised polymer attached through a steel alligator clip to tinned copper wire for solid state, a platinum wire auxiliary electrode and an electrolysed Ag/AgCl wire reference electrode separated from the solution by a CoralPor tip. Cyclic and differential pulse voltammograms were performed using a 10 mL solution of the analyte (1 mM complex in 0.1 M [(*n*-C_4_H_9_)_4_N]PF_6_ in either MeCN or DMF). The solution was purged with dried N_2_, Ar, or CO_2_ prior to each experiment. Ferrocene (Fc) (1 mM) was added as an internal standard during each experiment. All potentials are quoted in V *vs.* Fc^0^/Fc^+^. Uncompensated resistance between the working and the reference electrodes was corrected by using *i*_R_ compensation on the potentiostat. Scan rate dependence studies were carried out for each complex between 50–1600 mVs^−1^ to ensure the homogeneity of the system.

### Infrared spectroelectrochemistry (IR SEC)

Solution state IR SEC was performed on the discrete complexes using the IR-SEC cell previously reported by Kubiak *et al.*^41^ A Pine Instrument Co. Model AFCBP1 potentiostat was employed to control the cell potential, referenced to Ag/Ag^+^. Thin-layer bulk electrolysis was measured by reflectance IR off the electrode as the potential was scanned. All experiments were conducted in 0.1 M [(*n*-C_4_H_9_)_4_N]PF_6_/MeCN/DMF (9 : 1) with known analyte loadings prepared under an inert atmosphere. FT-IR spectra were recorded on a Thermo Scientific Nicolet 6700, with resolution of 4 cm^−1^. Unlike the CV or UV-Vis-NIR SEC experiments, it was not possible to use pure DMF in the IR SEC experiments, since the strong *ν*_C=O_ stretching vibration at 1740 cm^−1^ overwhelmed the comparatively weaker *ν*_C=N_ stretch expected from the salen metal complex at ∼1600 cm^−1^.

### Ultraviolet-visible-near infrared spectroelectrochemistry (UV-Vis-NIR SEC)

Solution state UV-Vis-NIR SEC was measured over the range 5000−35 000 cm^−1^ using a CARY5000 spectrophotometer interfaced to Varian WinUV software. The absorption spectra of the electrogenerated species were obtained *in situ* by the use of an Optically Semi-Transparent Thin-Layer Electrosynthetic cell, path length 0.685 mm, mounted in the path of the spectrophotometer. Solutions for the spectroelectrochemical experiment contained 0.1 M [(*n*-C_4_H_9_)_4_N]PF_6_/MeCN or [(*n*-C_4_H_9_)_4_N]PF_6_/DMF supporting electrolyte and *ca.* 0.4 mM of the compound for analysis. Appropriate potentials were applied by using an eDAQ e-corder 410 potentiostat and the current was carefully monitored throughout the electrolysis. The electrogenerated species formed *in situ*, and their absorption spectra were recorded at regular intervals through the electrolysis. The attainment of a steady-state spectrum and the decay of the current to a constant minimum at a potential appropriately beyond *E*_1/2_ (for the redox process) was indicative of the complete conversion of the starting material.

Solid state diffuse reflectance UV-Vis-NIR spectra of the redox-active species were collected *in situ* using a CARY5000 UV-Vis-NIR spectrophotometer equipped with a Harrick Omni Diff Probe attachment interfaced to Varian WinUV software over the range 5000–25 000 cm^−1^ in a custom-made cell previously reported by D'Alessandro *et al.*^42^ The cell consisted of a Pt wire counter electrode and a Ag/Ag^+^ quasi-reference electrode. The solid sample was immobilised by a thin strip of Teflon tape onto a 0.1 mm thick Indium-doped Tin Oxide (ITO) coated quartz slide, which functioned as the working electrode. The applied potential was controlled using an eDAQ potentiostat. Continuous scans of the sample were obtained and the potential increased gradually until a change in the spectrum was observed.

### Bulk electrolysis

Solution and solid state bulk electrolysis were performed in a threaded 60 mL single compartment cell with a custom airtight Teflon top, as reported by Kubiak *et al.*^43^ The set-up consisted of a carbon rod (surface area = 7.4 cm^2^) for solution state or glassy carbon plate with immobilised POP for solid state as the working electrode (active area = 2 cm^2^), a coiled Pt wire counter electrode protected from the bulk solution by fritted glass and an electrolysed Ag/AgCl pseudo reference, separated from solution by a CoralPor tip. The analyte solution (∼40 mL) consisted of complex (1–2 mM) in 0.1 M [(*n*-C_4_H_9_)_4_N]PF_6_/MeCN/DMF (8 : 2). The solution was purged with CO_2_ for 20 min prior to each electrolysis experiment, with the optimal applied potential determined by the CV experiments.

Gas phase analysis was performed by sampling 1 mL of the headspace of the cell at 20 min intervals and injecting into a Hewlett-Packard 7890A series gas chromatograph with two molecular sieve columns (30 m × 0.53 mm i.d. × 25 μm film). The 1 mL injection was split between two columns, one with N_2_ carrier gas and one with He carrier gas to quantify both CO and H_2_ respectively. Instrument specific calibration curves were measured prior to analysis to determine the amount of each gas produced.

Solution phase analysis was performed on the bulk solution to quantify formic acid production by sampling the bulk electrolysis solution (5 mL) after electrolysis. D_2_O (1 mL) was added to the solution and this was well mixed, prior to its dilution with dichloromethane. The D_2_O layer was separated prior to the addition of concentrated hydrochloric acid (1 drop). Samples were analysed by ^1^H NMR and spectra were recorded on a Bruker AVANCEIII 300 spectrometer operating at 300 MHz for ^1^H. Spectra were recorded at 298 K.

### Thermogravimetric analysis

TGA measurements were carried out on a TA Instruments Hi-Res 2950 Thermogravimetric Analyser or Discovery Thermogravimetric Analyser. Dry N_2_ (0.1 L min^−1^) flowed over the sample during data collection. The sample temperature was ramped at 1 °C min^−1^ from 25 to 600 °C. Samples were loaded dry after exposure to air.

### Gas adsorption

Adsorption isotherms were measured using the Accelerated Surface Area & Porosity (ASAP) 2020 or the 3-Flex, both supplied by Micromeritics Instruments Inc. The sample (∼50–100 mg) was loaded into a glass analysis tube and outgassed for 24 h under vacuum at 80 °C, prior to analysis.

N_2_ adsorption and desorption isotherms were measured at 77 K and data were analysed using the Brunauer, Emmett and Teller (BET) models to determine the surface area.^44^ Pore size distributions were calculated using the Density Functional Theory (DFT) cylindrical model in the Micromeritics MicroActive Software Package Version 4.03.

### Electrophoretic deposition (EPD) onto glassy carbon

Glassy carbon substrates were cut to 3 × 1 cm electrodes. The POP (20 mg) was suspended in toluene (20 mL) and sonicated for 30 s. Two identical glassy carbon substrates were dipped into the deposition solution (1 cm separation distance) and a constant DC voltage of 30 V was applied from an Extech 382270 High Precision Quad Output DC power supply. The deposition occurred over a period of 6 h, with a stirred suspension that was sonicated every 30 minutes to break up larger aggregates of POP. **Caution:** electrical sparking due to the accidental contact of electrodes and/or their leads can result in the spontaneous ignition of toluene. Prior to the undertaking of these experiments, ensure that the experiment is set up in an empty fume hood, clear of flammables and with a blast shield.

### Field emission scanning electron microscopy

FE-SEM measurements were obtained at either the Nano3 facility at The University of California, San Diego or at the Australian Centre for Microscopy & Microanalysis at The University of Sydney. POP materials were deposited onto a glassy carbon substrate, which was adhered to conductive carbon tape on a sample holder disk. The disk was coated using a Cr-sputter coating for 8 s. A Philips XL30 ESEM was used for acquiring images using a 10 kV energy source under vacuum at a working distance at 10 mm. ∼19 000× magnification images were collected.

## Results and discussion

### Synthesis and structural characterisation

**Scheme 1:**
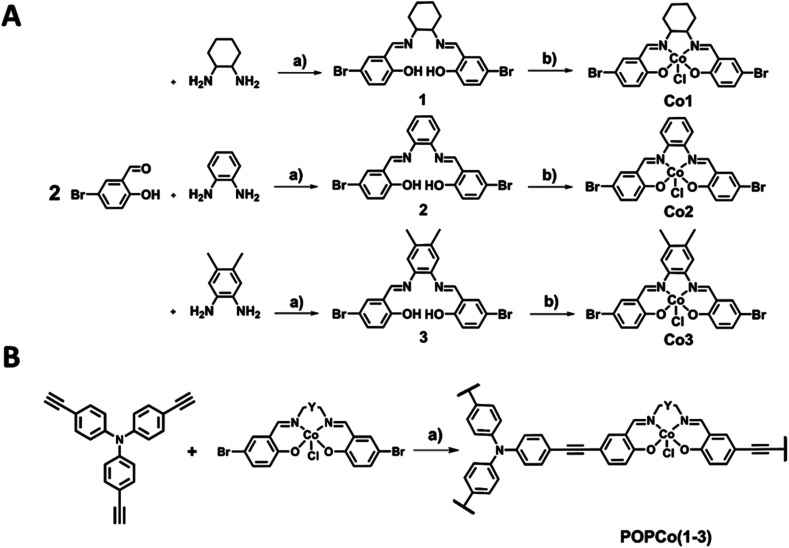
(A) Synthesis of bromo-terminated Co(iii) salen metal complexes (a) solvent: MeOH, 80 °C, 2 h, (b) metal: Co(OAc)_2_·4H_2_O (1.1 eq.), LiCl (4 eq.), solvent: EtOH, r.t., 48 h. (B) Synthesis of the Co(iii) salen polymer (a) catalysts: [Pd(PPh_3_)_4_] (30 mol%), CuI (30 mol%), solvent: toluene/EtOH (2 : 1), 85 °C, 72 h.

The synthesis of salen ligands was achieved by the Schiff-base condensation of 5-bromosalicylaldehyde with varying bridging diamines to afford the free-base salen in good yield. Co(iii) metalation was achieved by the addition of Co(OAc)_2_·4H_2_O in the presence of LiCl as an oxidising agent to afford all Co(iii) complexes in good yields (Scheme 1A). Salen metal complexes were successfully incorporated into POPs *via* a Sonogashira–Hagihara palladium cross coupling reaction between tris(*p*-ethynyl)triphenylamine (TPA) and the bis-bromo salen metal complexes under an inert N_2_ atmosphere in the presence of [Pd(PPh_3_)_4_] and CuI to form the series of POPs. In all reaction mixtures, the light yellow solution darkened and a brown precipitate formed (Scheme 1B).

Extensive characterisation was performed on the amorphous polymers to determine their composition. Despite their non-crystalline nature, TGA studies revealed that all POPs possessed thermal stability above 200 °C, with a number of polymers showing thermal stability of up to 400 °C, prior to partial thermal degradation (Fig. S1†). IR experiments indicated that the TPA co-ligand was covalently linked to the salen metal complex. The IR spectrum of TPA exhibited a *ν*_C≡H_ stretch at 3264 cm^−1^, which disappeared upon polymerisation. The weak *ν*_C≡C_ stretch also shifted from 2102 cm^−1^ in the monomer to 2191 cm^−1^ in the polymer.^45^ The *ν*_C=N_ stretch belonging to the imine in the salen metal complex was observed to shift upon incorporation into the salen POP compared with the polymer (Fig. S2†). Further evidence of the covalent linkage between co-ligand and salen was detected in the UV-Vis-NIR spectra of the monomers and polymers. The UV-Vis-NIR spectrum of TPA exhibited a sharp n–π* charge transfer at 26 460 cm^−1^ and π–π* charge transfer bands at 31 970 and 34 260 cm^−1^. When compared to the discrete salen complexes, many of the characteristic charge transfer bands in the salen metal complexes were shifted to lower energies in the polymers, consistent with polymeric systems that are more delocalised. The UV-Vis-NIR spectra of the polymers all exhibited the n–π* charge transfer band, consistent with TPA incorporation into the polymer (Fig. S3†). ICP-OES indicated that the Co(iii) salen was incorporated into the POP; however, the lower than calculated values for the Co(iii) content suggests that homocoupling between the TPA may be occurring because of uncontrolled propagation, as well as the possible leaching of the Co(iii) species (Table S1†).

**Fig. 2:**
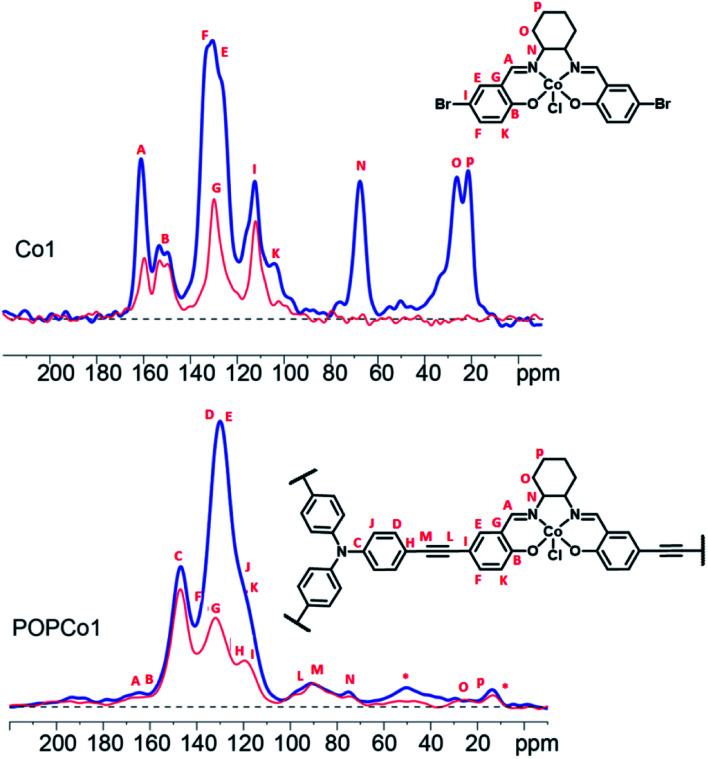
^13^C CPTOSS of Co1 (above) and POPCo1 (below). The full ^13^C NMR spectrum is plotted in blue, while the spectrum in red is the non-protonated methyl/carbon species detected after 40 μs of dipolar dephasing. The signals marked * are attributed to excess triethylamine, which was unable to be completely removed from the pores.

Solid state ^13^C NMR experiments were performed to further characterise the salen-based POPs. Solid state ^13^C NMR spectra could be obtained for POPCo1, POPCo2 and POPCo3, which all contain the Co(iii) d^6^ species. This implies that the Co(iii) salen metal complexes are low spin when incorporated into the POPs. The ^13^C CPMAS spectra of the polymers containing the diamagnetic salen metal complexes were collected and compared to the discrete complexes to show incorporation of the salen into the POP (Fig. 2, S4–S6†). A shift in the position of the alkynyl peaks from those observed in discrete TPA previously reported,^46^ as well as their broadening, suggests that a polymeric material has formed. Evidence for the incorporation of the salen metal complex came from the appearance of peaks from the bridging diamine around 160–165 ppm, corresponding to the imine carbon and phenolic carbon environments present in the salen. These varied dependent on the bridging moiety. The comparatively small signal intensity of the salen moiety suggests an incorporation of salen around 10%, which is consistent with the ICP-OES data (Table S1†). Variation in the incorporated salen metal complex could be deduced from the appearance of different carbon environments at lower field. For POPCo1, evidence for the diaminocyclohexane bridging moiety came from the appearance of peaks corresponding to the sp^3^ hybridised secondary and quaternary carbons on the bridging diamine between 20–30, and 70 ppm. The additional signals in the alkyl region at 15 and 50 ppm have been tentatively assigned to small quantities of residual triethylamine. For the aromatic POPCo2 and POPCo3, a larger integration under the aromatic signals from 110–160 ppm was detected, which implies additional carbon environments from the discrete complexes Co2 and Co3, respectively (Fig. S4 and S5†). Finally, the synthesis was repeated in the absence of salen metal complex, forming POPTPA. The ^13^C CPMAS spectrum for this deliberately homocoupled polymer POPTPA demonstrated the absence of peaks corresponding to the phenolic carbon and the imine of the salen moiety at 162–165 ppm. Additionally, a single ethynyl signal at 90 ppm was observed (Fig. S6†).

### Gas adsorption in polymers

Methods for the activation of POPs were initially probed as a step towards examining their gas sorption properties. The activation of the polymers was achieved by initially immersing the newly synthesised POP materials in DMF (10 mL) in an oven at 100 °C for 1 h, prior to filtration and repetition of the process three times. The polymer was subjected to washing *via* a Soxhlet washing process with methanol at 80 °C for 48 h.

**Fig. 3:**
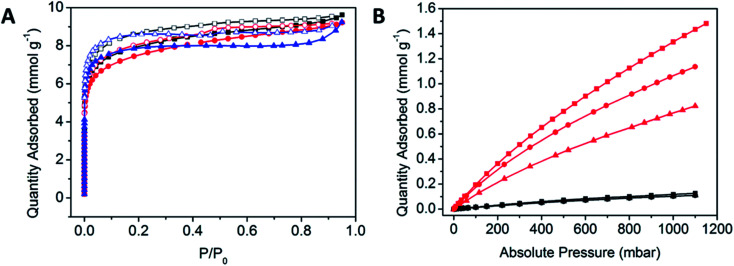
(A) N_2_ gas sorption isotherms at 77 K of POPCo1 (black), POPCo2 (red) and POPCo3 (blue). Closed squares represent N_2_ adsorption isotherms while open squares represent N_2_ desorption isotherms. (B) CO_2_ adsorption isotherms (red) and N_2_ gas adsorption isotherms (black) at 298 K of POPCo1 (circles), POPCo2 (triangles) and POPCo3 (squares).

The porosities of the POPs were analysed using N_2_ gas sorption experiments at 77 K to determine their BET surface areas. All POPs demonstrated Type I BET isotherm behaviour, which is indicative of a microporous material (Fig. 3A).^47^ There is a significant amount of hysteresis, which is commonly observed within highly flexible porous materials. The amorphous nature of the POPs is reflected in the pore size distribution, where there is a wide variety of sizes, consistent with the uncontrolled propagation of the POP (Table 1, Fig. S7†).

**Table 1:** BET surface areas, free pore volumes, pore size distributions, point selectivities and −*Q*_st_ of the synthesised polymers

Polymer	BET surface area (m^2^ g^−1^)	Pore volume (cm^3^ g^−1^)	Pore size distribution (Å)	Point selectivity *S*	−*Q*_st_ (kJ mol^−1^)
POPCo1	657 ± 1	0.36	4.9–7.7, 8.3–13	15.4	25–27
POPCo2	669 ± 1	0.37	4.8–7.8, 8.4–13, 17−21	9.5	27–29
POPCo3	688 ± 1	0.36	5.6–7.8, 8.8–12, 14−21	15.1	20–27

N_2_ and CO_2_ gas sorption experiments were performed at 298 K (Fig. 3B). The comparable N_2_ isotherms indicate that at 298 K, there is a limited uptake of N_2_ and a preference for CO_2_ uptake which could arise from differences in the salen backbone. Having to overcome the increased π-stacking from the aromatic POPCo2 may hinder the adsorption of CO_2_, while an increased bulk from the bridging diamine may disrupt these interactions, justifying why POPCo1 and POPCo2 have higher uptakes. The isosteric heats of adsorption indicate that there are physisorptive interactions between CO_2_ and the POP (Table 1, Fig. S8†). The slight variations in −*Q*_st_ suggest that the backbone of the salen does not play a significant role in the physisorptive interactions between CO_2_ and the POP. Finally, the CO_2_/N_2_ selectivities (*S*) were taken with respect to the conditions of a post-combustion flue stream at 298 K, consisting of N_2_ (*P*/*P*_0_ = 0.75) and CO_2_ (*P*/*P*_0_ = 0.15) (Table 1).

### Surface attachment of Co(iii) salen POPs to glassy carbon

**Fig. 4:**
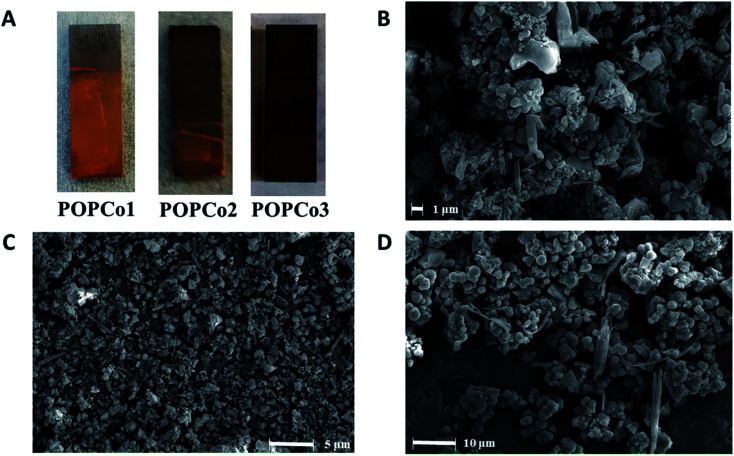
(A) Coverage of glassy carbon plates by Co(iii) polymers. FE-SEM images of (B) POPCo1 (C) POPCo2 and (D) POPCo3.

POPCo1, POPCo2 and POPCo3 were successfully immobilised onto glassy carbon *via* electrophoretic deposition by suspending the POP in toluene, with sonication and stirring while a constant DC voltage of 30 V was applied over a period of 6 h. The reaction was paused every hour to allow for the further sonication of the mixture to disrupt aggregation of the POP. The experiment resulted in coverage of POPCo1, POPCo2 and POPCo3, which could be imaged on the surface *via* FE-SEM (Fig. 4). POPCo1 and POPCo2 demonstrated better coverage of the glassy carbon plates than POPCo3.

### Electrochemistry

#### Discrete Co(iii) salen complexes

The three discrete Co(iii) complexes were first tested for their redox activity (Table 2, Fig. S9†). The CV experiment for Co1 revealed one irreversible redox process and two quasi-reversible processes. An additional reduction process could be observed upon inclusion of an aromatic bridging diamine, suggesting that the increased aromaticity of the salen improves its electronic properties. The addition of two methyl groups on the bridging diamine of Co3 shifted the reduction processes to more cathodic potentials relative to Co2, which was expected from a system containing increased electron density. The solution state CV of Co3 was akin to that of Co2.

**Table 2:** Redox features for Co(iii) salen metal complexes. Ferrocene (1 mM) was used as an internal standard. (0.1 M C_4_H_9_NPF_6_/DMF) was the supporting electrolyte. Where available, the peak–peak separation (Δ*E*) and the current ratio (*i*_pa_/*i*_pc_) of reversible and quasi-reversible redox features have been described

Complex	I_R_	II_R_	III_R_	IV_R_	Fc^0/+^
Co1	*E*_pc_ = −0.49 V	*E*_1/2_ = −1.66 V	*E*_1/2_ = −2.36 V		*E*_1/2_ = 0 V
		Δ*E* = 64 mV	Δ*E* = 60 mV		Δ*E* = 60 mV
		(*i*_pa_/*i*_pc_) = 0.642	(*i*_pa_/*i*_pc_) = 0.837		(*i*_pa_/*i*_pc_) = 0.914
Co2	*E*_pc_ = −0.37 V	*E*_1/2_ = −1.56 V	*E*_1/2_ = −2.23 V	*E*_1/2_ = −2.43 V	*E*_1/2_ = 0 V
		Δ*E* = 35 mV	Δ*E* = 63 mV	Δ*E* = 84 mV	Δ*E* = 61 mV
		(*i*_pa_/*i*_pc_) = 0.448	(*i*_pa_/*i*_pc_) = 0.597	(*i*_pa_/*i*_pc_) = 0.684	(*i*_pa_/*i*_pc_) = 0.944
Co3	*E*_pc_ = −0.39 V	*E*_1/2_ = −1.57 V	*E*_1/2_ = −2.25 V	*E*_1/2_ = − 2.46 V	*E*_1/2_ = 0 V
		Δ*E* = 33 mV	Δ*E* = 58 mV	Δ*E* = 59 mV	Δ*E* =62 mV
		(*i*_pa_/*i*_pc_) = 0.540	(*i*_pa_/*i*_pc_) = 0.705	(*i*_pa_/*i*_pc_) = 0.859	(*i*_pa_/*i*_pc_) = 0.918

#### Co(iii) salen POPs

Following the successful surface attachment of the Co(iii) salen POPs to glassy carbon, CV experiments were performed to examine their electronic behaviour. For the reduction sweeps, up to four redox processes were observed, corresponding to the reduction of the Co(iii) salen. There were fewer reduction processes observed for the aromatic systems. The irreversible III_R_ (in POPCo2 and POPCo3) and IV_R_ (in POPCo1) reduction process were assigned to the reduction of the imine across the salen metal complex. Incorporation of the salen into the POP appeared to bring the reduction potentials to more cathodic potentials, which is beneficial for any electrochemical applications (Table 3, Fig. S10†).

**Table 3:** Redox features for Co(iii) salen POPs. Ferrocene (1 mM) was used as an internal standard. 0.1 M LiBF_4_/CH_3_CN was the supporting electrolyte

POP	I_R_	II_R_	III_R_	IV_R_	Fc
POPCo1	*E*_pc_ = −0.12 V	*E*_1/2_ = −0.80 V	*E*_pc_ = −1.10 V	*E*_pc_ = −2.16 V	*E*_1/2_ = 0 V
POPCo2	*E*_pc_ = −1.42 V	*E*_pc_ = −2.35 V			*E*_1/2_ = 0 V
POPCo3	*E*_pc_ = −0.86 V	*E*_pc_ = −1.86 V	−2.26 V		*E*_1/2_ = 0 V

### Spectroelectrochemistry

#### Solution state SEC on Co(iii) salen complexes

**Scheme 2:**
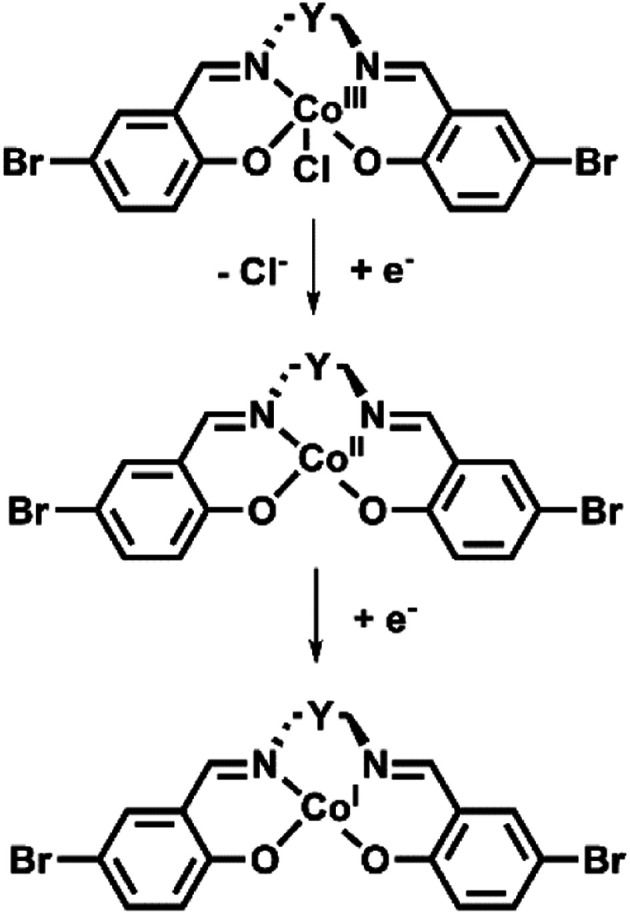
Proposed mechanism of the Co(iii) complexes observed over the initial two reduction processes.

For all discrete complexes, UV-Vis-NIR SEC experiments were performed to elucidate the mechanism for electron transfer (Scheme 2).

The first reduction processes in the complexes were consistent with the reduction of the Co(iii) centre to Co(i). In the SEC for all complexes, an increase in the intensity of the d–d bands (16 280 and 19 990 cm^−1^ for Co1, 18 570 cm^−1^ for Co2 and 15 040 cm^−1^ for Co3) was consistent with a transition from d^6^ to d^7^ cobalt species (Fig. S11†) and decreases in LMCT bands (24 470 in Co1, 20 980, 25 550 and 29 600 cm^−1^ in Co2 and 21 720, 29 550 and 30 980 cm^−1^ for Co3), as seen in other Co(iii) salen complexes.^48,49^ The maintenance of isosbestic points suggests that there is a clean conversion between the Co(iii), Co(ii) and Co(i) species.

**Fig. 5:**
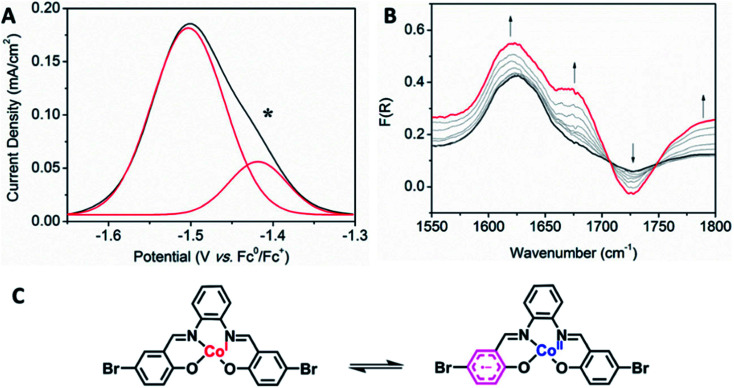
(A) Solution state DPV of Co2 (1 mM) (scan rate: 0.02 V s^−1^, Fc (1 mM) was used as an internal standard) and (B) solution state IR SEC of Co2 (4 mM) upon changing the potential from −0.8 to −1.5 V *vs.* Ag/Ag^+^. (0.1 M [(*n*-C_4_H_9_)_4_N]PF_6_/MeCN/DMF (9 : 1) as the supporting electrolyte). (C) The predicted mechanism of the II_R_ reduction process, showing possible charge transfer between the Co(i) metal and the salen backbone.

Interesting electronic properties appear after the II_R_ reduction. Solution state differential pulse voltammetry (DPV) experiments on Co2 in 0.1 M [(*n*-C_4_H_9_)_4_N]PF_6_/DMF under N_2_ reveal a non-Gaussian peak, indicating that the redox couple has a number of underlying processes (Fig. 5A). Despite multiple processes observed, the integration of the area under the combined processes is the same as the integration of the Fc^0^/Fc^+^ signal, indicating that only one electron is being transferred. UV-Vis-NIR SEC shows not only evidence for the Co(ii/i) couple (*vide supra*), but also an appearance of a radical band at 12 690 cm^−1^. IR SEC experiments were performed on Co2 (4 mM) in 0.1 M [(*n*-C_4_H_9_)_4_N]PF_6_/MeCN/DMF (9 : 1), which indicated that ligand-based processes are also occurring. The appearance of a new IR stretch at *ν* = 1674 cm^−1^, corresponding to a change in the *ν*_C–O_ stretch on the phenol, implies that there may be evidence of charge transfer from the Co(i) metal centre to the ligand (Fig. 5B).

**Fig. 6:**
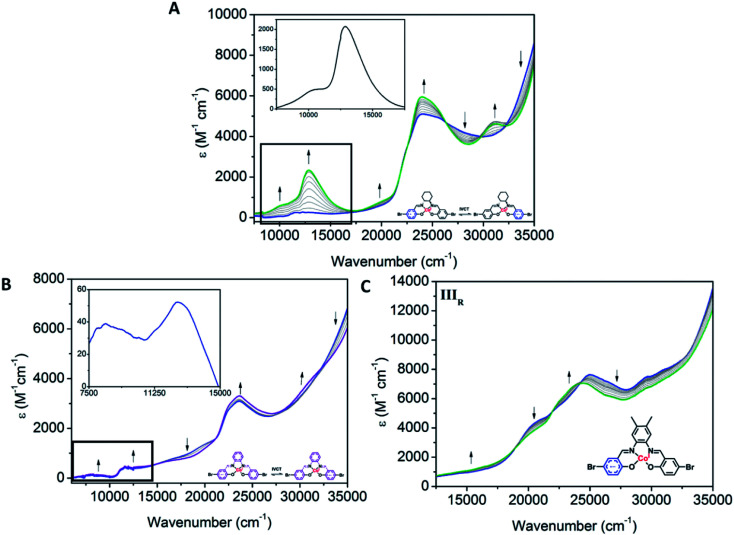
Solution state UV-Vis-NIR SEC of (A) Co1 (0.61 mM) upon changing the potential from −1.5 V to −1.8 V *vs.* Ag/Ag^+^, (B) Co2 (0.51 mM) upon changing the potential from −1.6 to −2.1 V *vs.* Ag/Ag^+^ and (C) Co3 (0.32 mM) upon changing the potential from −1.8 to −2.2 V *vs.* Ag/Ag^+^ (0.1 M [(*n*-C_4_H_9_)_4_N]PF_6_/DMF as the supporting electrolyte).

Clear differences in the ligand based reductions are also apparent between the materials. In Co1, the third reduction results in the generation of a number of important bands, consistent with intervalence charge transfer (IVCT). Upon application of a potential at *E*_pc_ = −1.66 V *vs.* Fc^0^/Fc^+^, the observation of a weak IVCT band at 10 010 cm^−1^ and a radical band at 12 860 cm^−1^ are suggestive of the formation of the salen radical anion. The lower energies associated with the radical anion band suggest that the Co(iii) metal centre promotes delocalisation. To the best of our knowledge, such IVCT transitions have not been previously reported for Co(iii) salen metal complexes. The appearance of the IVCT band is also accompanied by an increase in the π–π* transitions at 31 080 cm^−1^, consistent with improved electron delocalisation in the conjugated parts of the salen backbone. In Co2, there is evidence for a weaker IVCT transition. Changes appear in the LMCT and n–π* transitions at 19 590 and 23 680 cm^−1^, respectively. This is accompanied by the appearance of a weak IVCT band at 8340 cm^−1^ and a weak radical band at 12 670 cm^−1^. Similar processes in Co3 are observed. The dominant changes in the UV-Vis spectra are the decrease in the transitions at 20 500, 25 120, 29 550 and 30 980 cm^−1^, and the appearance of new transitions at 22 490 and 23 870 cm^−1^. This is also supported by the fact that the III_R_ process in Co3 is more cathodic than its Co2 analogue, which is consistent with improved delocalisation and electron density increasing the voltage required for reduction of the Co3 backbone. The absence of an observable IVCT band suggests that the degree of aromaticity affects the nature of the IVCT transition (Fig. 6).

#### Solid state SEC on Co(iii) salen polymers

**Fig. 7:**
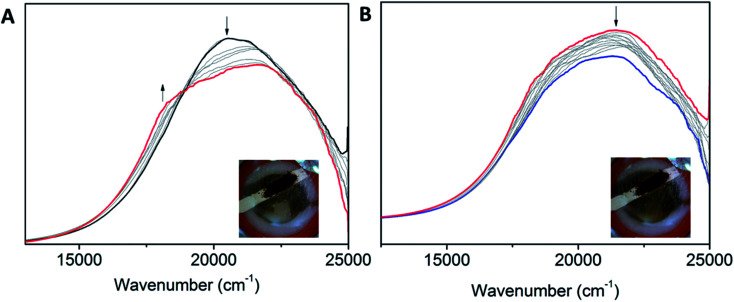
Solid state Vis-NIR SEC of POPCo3 upon changing the potential from (A) −1.0 to −1.5 V *vs.* Ag/Ag^+^, (B) −1.7 to −2.0 V *vs.* Ag/Ag^+^ (0.1 M [(*n*-C_4_H_9_)_4_N]PF_6_/MeCN as the supporting electrolyte).

*In situ* solid state SEC experiments were performed on POPCo1, POPCo2 and POPCo3. No significant changes were observed on the I_R_ reduction process of the polymer, corresponding to the loss of the chloride anion. Upon the application of a cathodic potential corresponding to the II_R_ redox potential in all systems, there was a darkening in the polymers that could be followed with change in the bands at 17 410 and 19 940 cm^−1^ (for POPCo1) (Fig. S12†) and 18 170 cm^−1^ and 20 510 cm^−1^ (for POPCo3) (Fig. 7). This change is consistent with a Co(ii/i) process and is reflected by corresponding changes in discrete complexes. There were small changes in the intensity of the peaks for POPCo2. Upon reaching the III_R_ potential, losses in intensity occurred in all of the bands in the spectra and the lack of stable isosbestic points indicated a degradation of the polymer. This is also evident from the change in the electrolyte solution from clear and colourless to green.

### Electrochemical behaviour in the presence of CO_2_

#### Co(iii) salen complexes

**Fig. 8:**
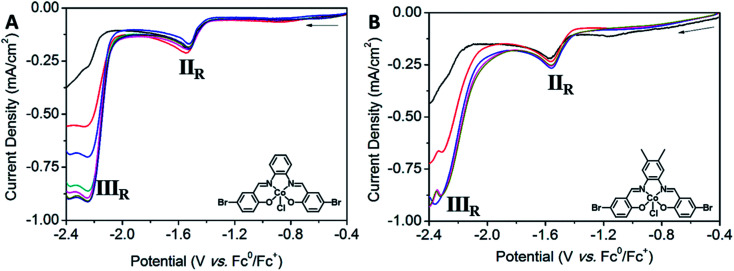
Solution state CV showing the electrochemical response of (A) Co2 (1 mM) under saturation of N_2_ (black), CO_2_ (red) and CO_2_ with TFE (0.7 mmol-blue, 1.4 mmol-green, 2.1 mmol-light pink, 2.8 mmol-pink, 3.5 mmol-brown) and (B) Co3 (1 mM) under saturation of N_2_ (black), CO_2_ (red) and CO_2_ with TFE (0.7 mmol-blue, 1.4 mmol-pink). Return sweeps are not shown for clarity (0.1 M [(*n*-C_4_H_9_)_4_N]PF_6_/DMF as the supporting electrolyte, scan rate: 0.1 V s^−1^, Fc (1 mM) was used as an internal standard).

Co(iii) salen complexes were evaluated for their electrochemical behaviour in the presence of CO_2_. For Co1, there was a slight increase in the magnitude of the current density of II_R_ for Co1, indicating that it was not fit for CO_2_ reduction (Fig. S13A†). The titration of TFE into the solution did not enhance with the current; therefore, further electrochemical measurements were not pursued. More pronounced changes in the presence of CO_2_ were observed in Co2, where the I_O_ redox process at *E*_pa_ = +0.37 V *vs.* Fc^0^/Fc^+^ disappeared, with changes observed in the I_R_ redox process at *E*_pc_ = −0.37 V *vs.* Fc^0^/Fc^+^ (Fig. S13B†). A slight anodic shift was observed for the II_R_ redox process at *E*_1/2_ = −1.56 V *vs.* Fc^0^/Fc^+^, corresponding to the Co(ii/i) reduction. This may indicate weak association between the reduced species and CO_2_. The III_R_ process at *E*_1/2_ = −2.23 V *vs.* Fc^0^/Fc^+^ became significant under the CO_2_ atmosphere and continued to increase with the addition of aliquots of TFE, indicating that a proton source was required to enhance activity. An absence of this increase under N_2_ with TFE indicates that the process is not only related to the proton reduction of H_2_, but is likely to arise from the reduction of CO_2_. The reduction of CO_2_ by Co2 follows pseudo-first order kinetics. A plot of *i*_cat_ against the square root of [CO_2_] revealed a linear relationship between these two variables, indicating that the process is first order in CO_2_ (Fig. S14A†). The plot of *i*_cat_*vs.* [H^+^] showed an initial second order dependence on the proton source, reaching a maximum current density at 0.90 mA cm^−2^ after 0.15 M TFE (Fig. S14B†). Plotting *i*_cat_*vs.* [analyte] revealed a linear relationship (Fig. S14C†). The concentration of CO_2_ (0.23 M in DMF) was well in excess of the concentration of the analyte (1 mM). The titration of TFE into the reaction mixture under an atmosphere of CO_2_ resulted in the increase in the current density of the III_R_ redox process at *E*_pc_ = −2.23 V *vs.* Fc^0^/Fc^+^ to a maximum. The addition of TFE into the reaction mixture resulted in a peak ratio 
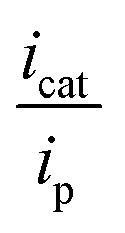
 of 3.34, corresponding to a TOF *k*[Q] of 2.16 s^−1^ at 0.63 M TFE. Only a weak association of CO_2_ with Co2 was observed (Fig. 8).

Solution state CV experiments performed on Co3 in the presence of CO_2_ resulted in a slight anodic shift in the II_R_ redox process at *E*_1/2_ = −1.57 V *vs.* Fc^0^/Fc^+^, corresponding to the Co(ii/i) process and indicating a weak association of CO_2_ to the metal centre. The III_R_ redox process at *E*_1/2_ = −2.25 V *vs.* Fc^0^/Fc^+^ increased under CO_2_ saturation, and was further enhanced to a maximum upon the addition of successive aliquots of TFE, indicating that a proton source is required to achieve optimal activity. The absence of this enhancement under N_2_ with the presence of TFE indicates that the process is not solely related to proton reduction, but is more likely to arise from the reduction of CO_2_. Like Co2, Co3 also follows pseudo first order kinetics. A plot of *i*_cat_ against the square root of [CO_2_] showed a linear relationship, suggesting first order kinetics in [CO_2_] (Fig. S15A†). The plot of *i*_cat_*vs.* [H^+^] shows an initial second order dependence on TFE, prior to reaching saturation (Fig. S15B†). In Co3, saturation of the proton source occurs at lower concentrations than its Co2 analogue, implying that a lower concentration of a proton source facilitates the reduction of CO_2_. Plotting *i*_cat_*vs.* [analyte] reveals a linear relationship, indicating a first order dependence of peak current on [analyte] (Fig. S15C†). CO_2_ (0.23 M in DMF) is well in excess of the analyte. Solution state CV experiments were performed on Co3 (1 mM) with varying concentrations of TFE titrated into the solution. The titration of TFE into the reaction mixture under an atmosphere of CO_2_ resulted in an increase in the current density of the III_R_ process to a maximum at *E*_pc_ = −2.25 V *vs.* Fc^0^/Fc^+^ after only two aliquots. Addition of excess TFE after this point resulted in no significant change. The addition of TFE into the reaction mixture yielded a peak ratio 
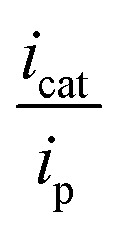
 of 2.98, corresponding to a TOF *k*[Q] of 1.72 s^−1^ at 0.14 M TFE, which is a smaller turnover than the Co2 analogue. A negligible association with CO_2_ was observed.

CPE experiments were performed on solutions of both Co2 (3.60 mM) with TFE (0.63 M) and Co3 (1.11 mM) with TFE (0.14 M) in 0.1 M [(*n*-C_4_H_9_)_4_N]PF_6_/DMF/MeCN (8 : 2) under CO_2_. The generation of CO was observed in the gas phase from both complexes, as measured from the first 3.73 turnovers over 3 h in Co2 and measured from the first 4.15 turnovers over the space of 4 h in Co3 (Table 4). A linear relationship in the production of gas was maintained throughout the duration of the CPE experiments for both complexes, indicating that the true lifetime of the catalyst may be slightly longer, although there is evidence for both H_2_ and CO production plateauing (Fig. S16†). The analyte sustained current densities of 2.57 mA cm^−2^ for Co2 and 0.4 mA cm^−2^ for Co3, with CPE experiments revealing a sustained current density over the period of the transformation. This suggests that the electrochemical transformation of CO_2_ is more likely to be stoichiometric rather than catalytic.

**Table 4:** Faradaic efficiencies for the generation of H_2_ and the conversion of CO_2_ into CO and HCOOH. All experiments were performed in (0.1 M [(*n*-C_4_H_9_)_4_N]PF_6_/DMF/MeCN (8 : 2) with TFE as the supporting electrolyte under CO_2_, *E*_pc_ = −1.85 V *vs.* Ag/Ag^+^)

Experiment	*F*_CO_ (%)	*F*_H_2__ (%)	*F*_HCOOH_ (%)
Solvent	0	0	0
Solvent + TFE (0.7 M)	0	12	0
Co2 +TFE (0.63 M)	20	13	7
Co3 + TFE (0.14 M)	11	14	0

The solution of the electroanalyte was analysed *via*^1^H NMR experiments for solution phase products post electrolysis. After aqueous work up into D_2_O, ^1^H NMR revealed a peak at *δ* = 8.00 ppm, corresponding to the generation of formic acid (Fig. S17†). An NMR time-course experiment was performed concurrently with CPE experiments on Co2, which demonstrated that the peak increased with the CPE. Broadening of the peak may be indicative of paramagnetic metal ions leaching into the solution. In the absence of CO_2_, the peak corresponding to the formation of formic acid did not appear. No formic acid was observed during experiments where CO_2_ was present but Co2 was absent, indicating that the transformation of CO_2_ into formic acid is due to both the presence of CO_2_ and Co2.

The prime difference between the analytes of Co2 and Co3 lies in the generation of both gaseous phase and solution phase products from the reduction of CO_2_. Although CO was formed from both complexes, it was formed in lower efficiencies from the more electron donating Co3. Following the CPE experiments on Co3, the electroanalyte was analysed *via*^1^H NMR experiments after work up into D_2_O. No evidence for formic acid was found, which may imply that selectivity for the generation of CO over formic acid could be achieved by varying the electronics of the salen backbone. Future studies will require the analysis of more electron withdrawing species and their effect on the reduction of CO_2_.

#### Co(iii) salen POPs

Solid state CV measurements were performed on the polymer series in the presence of CO_2_. For POPCo1 and POPCo2, there was a loss in intensity of all redox processes, suggesting that CO_2_ switches off the redox activity of the POP (Fig. S18†). Detailed CO_2_ reduction experiments were therefore not pursued. Significant changes were observed in the solid state CV of POPCo4. Compared to the CV under the N_2_ atmosphere, a decrease in intensity of the II_R_ and III_R_ processes was observed, as well as an anodic shift of the II_R_ process from *E*_pc_ = −1.86 V to −1.73 V *vs.* Fc^0^/Fc^+^, indicating that CO_2_ could be irreversibly binding to the species formed from the II_R_ process. The II_R_ process increased to a maximum upon the titration of successive aliquots of TFE. This was accompanied by the generation of a new irreversible process N_1_ at *E*_pc_ = −1.96 V *vs.* Fc^0^/Fc^+^. A CPE experiment was performed in 0.1 M LiBF_4_/MeCN with TFE (0.5 M) at *E*_pc_ = −2.00 V *vs.* Fc^0^/Fc^+^ under CO_2_ saturation. Over the space of 3 h, the initial current decreased, before reaching a maximum of 0.67 mA cm^−2^ after 1 h. Solution and gas phase analysis were unable to detect products of CO_2_ reduction, which may be due to the micromolar concentration of Co(iii) metal sites in the polymer in the electrochemical cell and the detection limits of the GC and NMR. This may also suggest degradation of the polymer under electrochemical stimulus.

## Conclusions

This work sought to explore the use of Co(iii) salen metal complexes and POPs as agents for the electrochemical reduction of CO_2_. A series of discrete Co(iii) bis-bromo terminated salen metal complexes were synthesised and characterised using NMR, UV-Vis-NIR, IR, CV and various SEC techniques to understand their physical and electrochemical properties. The two salen metal complexes, Co2 and Co3, successfully reduced CO_2_ to CO in 20% and 11% Faradaic efficiencies, respectively, suggesting scope to use these complexes as multi-electron reducing agents. Formic acid was also observed as a product of CO_2_ reduction in Co2 but not in Co3, implying that a more electron poor salen metal complex improves the conversion of CO_2_ into commodity chemicals. Further functionalisation of the bridging diamine may allow for the selectivity of products to be tuned. This work has shown the potential for expanding the studies of Co(iii) salen complexes as reducing agents for two-electron transfer reactions; however, additional work is required to improve the stability of the electrogenerated imine radical that forms.

The discrete salen complexes were successfully incorporated into POP materials through the Sonogashira–Hagihara reaction with TPA to generate POPCo1, POPCo2 and POPCo3, and were characterised by UV-Vis-NIR, IR, elemental analysis, FE-SEM, solid state NMR and gas sorption. The polymers were found to be porous, with BET surface areas up to 690 m^2^ g^−1^ (comparable with those reported in the literature).^21,27,49^ The successful reduction of CO_2_ into CO and formic acid by the discrete complexes led to the examination of the electrochemical properties of the POPs through solid state CV, CPE and SEC experiments. It was possible to use mild EPD conditions to adhere the POP materials to the conductive glassy carbon surface as a thin film, which could be characterised through FE-SEM. Solid state CV experiments revealed a number of irreversible processes for these polymers, suggesting that the instability of the POP material for electrochemical purposes arises from the imine core. When exposed to CO_2_, the redox behaviour was turned off, suggesting that there is an irreversible chemical transformation of CO_2_ in the polymer, which could be confirmed from Vis-NIR SEC. There is further scope to address this shortcoming by reinforcing the stability of the imine.

## Conflicts of interest

The authors declare no conflicts of interest.

## Supplementary Material

RA-008-C8RA04385J-s001
