# Supplementary material for: Electroactive Co(iii) salen metal complexes and the electrophoretic deposition of their porous organic polymers onto glassy carbon
Source: RSC Adv. 2018 Jul 3;8(43):24128–42. doi: 10.1039/c8ra04385j (PMC9081851; doi:10.1039/c8ra04385j)
Supplement: RA-008-C8RA04385J-s001 [file RA-008-C8RA04385J-s001.pdf]

Electronic Supplementary Material (ESI) for RSC Advances.
This journal is © The Royal Society of Chemistry 2018

Electroactive Co(III) Salen Metal Complexes and the Electrophoretic Deposition of their Porous Organic Polymers onto Glassy Carbon

Marcello B. Solomon,[a] Aditya Rawal,[b] James M. Hook,[b] Seth M. Cohen,[c] Clifford P. Kubiak,[c] Katrina A. Jolliffe,*[a] Deanna M. D'Alessandro*[a]

[a] *School of Chemistry, The University of Sydney, New South Wales 2006, Australia, Fax: +61 2 9351 3329; Tel: +61 2 9351 3777; E-mail: deanna.dalessandro@sydney.edu.au; kate.jolliffe@sydney.edu.au*

[b]*NMR Facility, Mark Wainwright Analytical Centre, The University of New South Wales 2052, Australia*

[c]*Department of Chemistry and Biochemistry, University of California, San Diego, California 92093, United States*

Synthesis

The synthesis of *tris*(p-ethynylphenyl)amine (**TPA**) was carried out according to the literature procedure.[1] The *tetrakis*(triphenylphosphine) palladium(0) catalyst [Pd(PPh_3)$_4$] was synthesised from palladium(II) chloride (Precious Metals Online) according to the literature procedure.[2] The synthesis of the free base ligands 2, 2ˈ-[(1R,2R)-1,2-Cyclohexanediylbis(nitrilomethylidine)]bis[4-bromo-phenol] (**1**),[3] 2, 2ˈ-[1,2-Phenylenediyl bis(nitrilomethylidine)]bis[4-bromo-phenol] (**2**),[4] and 2, 2ˈ-[4,5-Dimethyl-1,2-phenylenediyl bis(nitrilomethylidine)]bis[4-bromo-phenol] (**3**) [5] have previously been reported and their synthetic methods were adapted.

Salen backbones

2, 2ˈ-[(1R,2R)-1,2-Cyclohexanediylbis(nitrilomethylidine)]bis[4-bromo-phenol] (1).

5-Bromosalicylaldehyde (1.20 g, 6.00 mmol) was dissolved in degassed MeOH (10 mL). (1*R*,2*R*)-diaminocyclohexane (0.332 g, 3.00 mmol) was added, upon which the solution immediately turned bright yellow. The reaction was heated at 80 °C for 2 h. The bright yellow precipitate was separated from the mixture *via* centrifugation and the yellow solid was washed with cold MeOH (3 × 3 mL) and dried to yield a bright yellow powder (Yield: 1.13 g, 79%). **M.P.** 185–187 °C (lit.[6] 186–188 °C) **^{1}H NMR** (200 MHz, d_6-DMSO) δ (ppm) 13.36 (s, 1H), 8.48 (s, 1H), 7.59 (d, $^4J_{H\text{-}H}$ = 2.6 Hz, 1H), 7.41 (dd, $^3J_{H\text{-}H}$ = 8.9 Hz, $^4J_{H\text{-}H}$ = 2.6 Hz, 1H), 6.80 (d, $^3J_{H\text{-}H}$ = 8.9 Hz, 1H), 3.39–3.45 (m, 1H), 1.35–1.98 (m, 2H). The characterisation data matched that reported in the literature.[3]

2, 2ˈ-[1,2-Phenylenediyl bis(nitrilomethylidine)]bis[4-bromo-phenol] (2).

5-Bromosalicylaldehyde (0.600 g, 3.00 mmol) was dissolved in degassed MeOH (10 mL). *o*-Phenylenediamine (0.162 g, 1.50 mmol) was added, upon which the solution turned bright orange immediately. The reaction was heated at 80 °C for 2 h. The bright orange precipitate was separated from the reaction mixture *via* centrifugation and the solid was washed with cold ether (3 × 5 mL) and dried to yield a bright orange powder (Yield: 0.614 g, 87%). **M.P.** 216–217 °C (lit.[7] 216–218 °C) **^{1}H NMR** (400 MHz, d_6-DMSO) δ (ppm) 12.90 (s, 1H), 8.91 (s, 1H), 7.89 (d, $^4J_{H\text{-}H}$ = 2.6 Hz, 1H), 7.54 (dd, $^3J_{H\text{-}H}$ = 8.8 Hz, $^4J_{H\text{-}H}$ = 2.6 Hz, 1H), 7.40–7.48 (m, 2H), 6.94 (d, $^3J_{H\text{-}H}$ = 8.8 Hz, 1H). The characterisation data matched that reported in the literature.[4]

2, 2′-[4,5-Dimethyl-1,2-phenylenediyl bis(nitrilomethylidine)]bis[4-bromo-phenol] (3).

5-Bromosalicylaldehyde (0.600 g, 3.00 mmol) was dissolved in degassed MeOH (10 mL). 4,5-Dimethyl-*o*-phenylenediamine (0.204 g, 1.50 mmol) was added to the solution, which turned bright orange immediately. The reaction was heated at 80 °C for 2 h. The precipitate that formed was separated from the reaction mixture *via* centrifugation and was washed with cold ether (3 × 5 mL) and dried to yield a bright orange powder (Yield: 0.741 g, 98%). **M.P.** 241–243 °C (lit.[5] 241–245 °C) **^{1}H NMR** (400 MHz, d_6-DMSO) δ (ppm) 8.91 (s, 1H), 7.87 (d, $^4J_{H\text{-}H}$ = 2.5 Hz, 1H), 7.52 (dd, $^3J_{H\text{-}H}$ = 8.8 Hz, $^4J_{H\text{-}H}$ = 2.5 Hz, 1H), 7.28 (m, 1H), 6.92 (d, $^3J_{H\text{-}H}$ = 8.8 Hz, 1H), 2.30 (s, 3H). The characterisation data matched that reported in the literature.[5]

Co(III) salen metal complexes.

2, 2′-[(1R,2R)-1,2-Cyclohexanediylbis(nitrilomethylidine)]bis[4-bromo-phenol]Co(III) chloride (Co1).

EtOH (50 mL) was degassed with N_2 for 20 minutes. Under N_2, **1** (0.480 g, 1.00 mmol) was dissolved in EtOH and to this solution was added $Co(OAc)_2$•$4H_2O$ (0.273 g, 1.10 mmol), upon which the solution turned brown. The solution was stirred at room temperature for 2 h, prior to the removal of the N_2 atmosphere and the addition of LiCl (0.187 g, 4.41 mmol). The mixture was allowed to stir in air at room temperature for 2 d. The excess EtOH was removed *in vacuo* and the residue dissolved in DCM (150 mL) and washed with H_2O (3 × 50 mL). The organic layer was collected, dried over Na_2SO_4 and evaporated to yield the product as a dark brown powder (Yield: 0.544 g, 95%) **M.P.** 297–299 °C **ATR-IR** (cm^{-1}) $\nu_{C=N}$ = 1638 **UV-Vis-NIR** λ(KBr, cm^{-1}) 12435, 17886 (sh), 25002, 30029, 34708 **MS (ESI+, DMF/MeOH)** *m/z* calculated for $C_{20}H_{18}Br_2CoN_2O_2$ [M-Cl]$^+$: 537.12, found: 536.93 **Elemental Analysis** calculated for $C_{20}H_{18}Br_2ClCoN_2O_2$: C 42.0, H 3.2, N 4.9%, found C 41.6, H 3.2, N 4.8%.

2, 2′-[1,2-Phenylenediyl bis(nitrilomethylidine)]bis[4-bromo-phenol]Co(III) chloride (Co2).

EtOH (50 mL) was degassed with N_2 for 20 min. Under an atmosphere of N_2, **2** (0.348 g, 0.735 mmol) was dissolved in EtOH and $Co(OAc)_2$•$4H_2O$ (0.202 g, 0.810 mmol) was added to this solution, upon which the solution turned brown. The solution was stirred at room temperature for 2 h, prior to the removal of N_2 and the addition of LiCl (0.136 g, 3.21 mmol). The mixture was allowed to stir in air at room temperature for 2 d. The excess EtOH was removed and the residue washed with H_2O (30 mL), EtOH (30 mL) and diethyl ether (30 mL) to yield the product as a dark brown powder (Yield: 0.314 g, 76%) **M.P.** 330–333 °C **ATR-IR** (cm^{-1}) $\nu_{C=N}$ = 1613 **UV-Vis-NIR** λ(KBr, cm^{-1}) 12154 (sh), 16485 (sh), 20187, 21376, 30377 **MS (ESI+, DMF/MeOH)** *m/z* calculated for $C_{20}H_{12}Br_2CoN_2O_2$ [M-Cl]$^+$: 531.07, found: 530.93 **Elemental Analysis** calculated for $C_{20}H_{18}Br_2ClCoN_2O_2$: C 42.4, H 2.1, N 4.9%, found C 42.1, H 2.1, N 4.8%.

2, 2′-[4,5-Dimethyl-1,2-phenylenediyl bis(nitrilomethylidine)]bis[4-bromo-phenol] (Co3).

EtOH (20 mL) was degassed with N_2 for 20 min. Under an atmosphere of N_2, **3** (0.250 g, 0.500 mmol) was dissolved in EtOH and $Co(OAc)_2$•$4H_2O$ (0.127 g, 0.510 mmol) was added to this solution, upon which the solution turned brown. The solution was stirred at room temperature for 2 h, prior to the removal of N_2 and the addition of LiCl (0.086 g, 2.04 mmol). The mixture was allowed to stir in air at room temperature for 2 d. The excess EtOH was removed and the residue was washed with H_2O (30 mL), EtOH (30 mL) and diethyl ether (30 mL) to yield the product as a dark brown powder (Yield: 0.256 g, 87%) **M.P.** > 350 °C **ATR-IR** (cm^{-1}) $\nu_{C=N}$ = 1622 **UV-Vis-NIR** λ(KBr, cm^{-1}) 14948 (sh), 21928,

25426, 29681 **MS (ESI+, DMF/MeOH)** *m/z* calculated for $C_{22}H_{16}Br_2CoN_2O_2$ $[M-Cl]^+$: 558.89, found: 558.80 **Elemental Analysis** calculated for $C_{20}H_{18}Br_2ClCoN_2O_2$: C 44.4, H 2.7, N 4.7%, found C 44.7, H 3.0, N 4.8%.

POPs containing Co(III) salen complexes.

General procedure for the synthesis of POPs.

A solution of toluene/EtOH (2:1) was degassed with N_2 for 20 min. The *bis*-bromo salen metal complex, **TPA**, $[Pd(PPh_3)_4]$ were suspended in the degassed solution and stirred away from light for 15 min. CuI was added and the reaction heated at 85 °C for 72 h. The solid was collected by filtration, washed with DMF (100 mL), toluene (100 mL), MeOH (100 mL) and extracted *via* a Soxhlet washing procedure with MeOH for 48 h. The fluffy brown solid was dried under vacuum overnight.

POPCo1.

Co1 (0.115 g, 0.202 mmol), **TPA** (0.255 g, 0.804 mmol), $[Pd(PPh_3)_4]$ (0.080 g, 0.069 mmol) and CuI (0.040 g, 0.210 mmol) were all combined in solution (15 mL) and reacted according to the general procedure to afford the polymer (Yield: 0.312 g) **M.P.** > 350 °C **ATR-IR** (cm^{-1}) $\nu_{C\equiv C}$ = 2192, $\nu_{C=N}$ = 1595 **UV-Vis-NIR** λ(KBr, cm^{-1}) 16050, 22280, 30520 **Elemental Analysis** Found: C 73.8, H 4.6, N 4.3%.

POPCo2.

Co2 (0.113 g, 0.200 mmol), **TPA** (0.255 g, 0.804 mmol), $[Pd(PPh_3)_4]$ (0.080 g, 0.069 mmol) and CuI (0.040 g, 0.210 mmol) were all combined in solution (15 mL) and reacted according to the general procedure to afford the polymer (Yield: 0.209 g). **M.P.** > 350 °C **ATR-IR** (cm^{-1}) $\nu_{C\equiv C}$ = 2195, $\nu_{C=N}$ = 1592 **UV-Vis-NIR** λ(KBr, cm^{-1}) 16720, 22350, 31320 **Elemental Analysis** Found: C 70.7, H 4.3, N 4.6%.

POPCo3.

Co3 (0.120 g, 0.203 mmol), **TPA** (0.255 g, 0.804 mmol), $[Pd(PPh_3)_4]$ (0.080 g, 0.069 mmol) and CuI (0.040 g, 0.210 mmol) were all combined in solution (15 mL) and reacted according to the general procedure to afford the polymer (Yield: 0.290 g). **M.P.** >350 °C **ATR-IR** (cm^{-1}) $\nu_{C\equiv C}$ = 2203, $\nu_{C=N}$ = 1595 **UV-Vis-NIR** λ(KBr, cm^{-1}) 15780, 20940, 30120, 34870 **Elemental Analysis** Found: C 75.6, H 4.4, N 4.3%.

POPTPA.

TPA (0.200 g, 0.630 mmol), $[Pd(PPh_3)_4]$ (0.080 g, 0.069 mmol) and CuI (0.040 g, 0.210 mmol) were combined in solution (15 mL) and reacted according to the general procedure to afford the polymer (Yield: 0.136 g). **M.P.** >350 °C **ATR-IR** (cm^{-1}) $\nu_{C\equiv C}$ = 2195, **UV-Vis-NIR** λ(KBr, cm^{-1}) 21540, 32400 **Elemental Analysis** Found: C 71.0, H 4.0, N 3.6%.

Calculations

If an electrochemical system obeys pseudo-first order kinetics (proof that the reaction is first order in the analyte and the concentration of CO_2, and that the concentration of CO_2 is large in comparison to that of the analyte), it is possible to calculate kinetic data for the interaction of salen complexes with CO_2. Upon comparison of the peak currents for salen complexes in the presence of CO_2 with the absence of CO_2, an expression for $k_{cat}[Q]$, or TOF, in terms of the $\frac{i_{cat}}{i_p}$ ratio, which can be directly examined from CV.

$$k[Q] = \frac{Fvn_p^3}{RT}\left[\frac{0.4463}{n_{cat}}\right]^2\left[\frac{i_{cat}}{i_p}\right]^2 \tag{1}$$

where F = Faraday constant (C mol^{-1}), ν = scan rate (V s^{-1}), n_p = number of electrons facilitated by the redox process, R = universal gas constant (J mol^{-1} K^{-1}), T = temperature (K), n_{cat} = number of electrons facilitated in the catalytic transformation, i_{cat} = peak catalytic current (mA/cm^2), i_p = peak current under N_2 (mA/cm^2).

Low pressure CO_2 measurements (up to 1 bar) were carried out at three temperatures (typically 288, 298 and 308 K) on the ASAP2020 or 3-Flex as described above. The data were modelled using a virial equation or the interpolation function before applying the Clausius-Clapeyron relation. The heat of adsorption for CO_2 was determined by comparing CO_2 isotherms at 288, 298 and 308 K. Isosteric heat of adsorption calculations (Q_{st}) for CO_2 at these temperatures were undertaken using the Clausius-Clapeyron equation

$$(\ln P)_n = -\left(\frac{Q_{st}}{R}\right)\left(\frac{1}{T}\right) + C \tag{2}$$

where P = pressure (mbar), n = amount of gas adsorbed (mol/mol), T = temperature (K), R = universal gas constant (J mol^{-1} K^{-1}) and C = constant).

The selectivity (S) for adsorption of CO_2 over N_2 was estimated from the single-component N_2 and CO_2 room temperature isotherm data. The values for this approximation are derived from an approximate flue gas composition of 15% CO_2, 75% N_2 and 10% other gases, at a total pressure of 1 bar.

$$S = \frac{\left(\frac{q_{CO_2}}{q_{N_2}}\right)}{\left(\frac{p_{CO_2}}{p_{N_2}}\right)} \tag{3}$$

where q = quantity of gas adsorbed (mmol g^{-1}), p = partial pressure at which each gas is adsorbed).

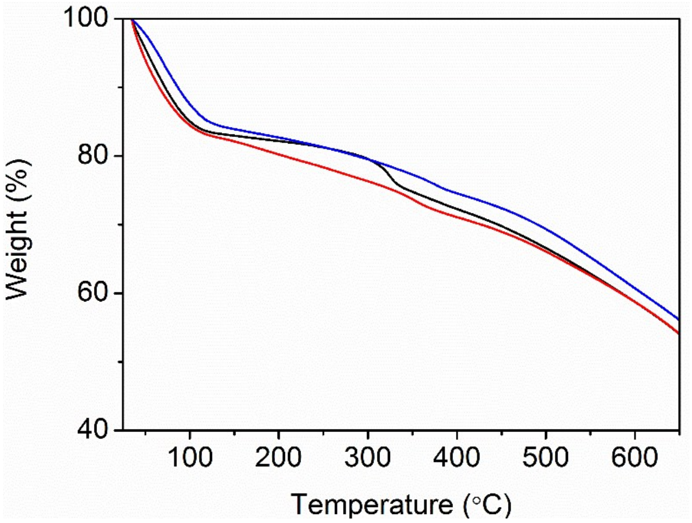

Figure S1: TGA of **POPCo1** (black), **POPCo2** (red) and **POPCo3** (blue) taken from 25 to 650 °C. The temperature was ramped at 1 °C min^{-1}.

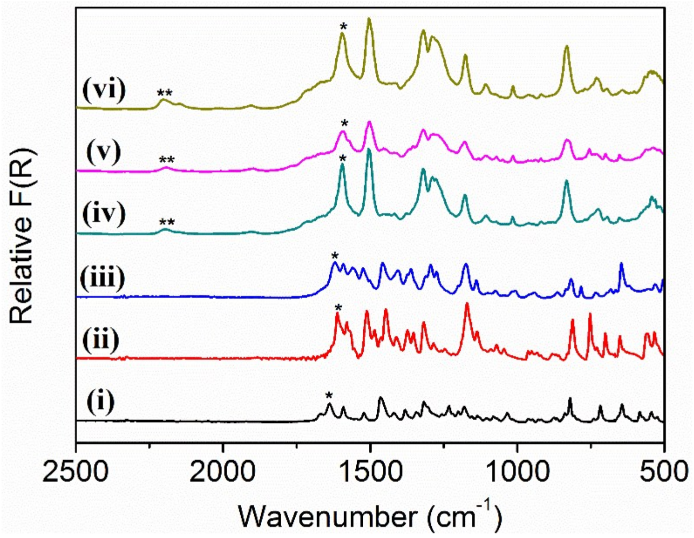

Figure S2: Solid State ATR-IR measurements of **(i) Co1 (ii) Co2 (iii) Co3 (iv)POPCo1 (v) POPCo2** and **(vi) POPCo3**. * denotes the shift in the $\nu_{C=N}$ stretch from the discrete complexes to the POPs, while the $\nu_{C\equiv C}$ stretch appears in the polymers but not in the discrete complexes.

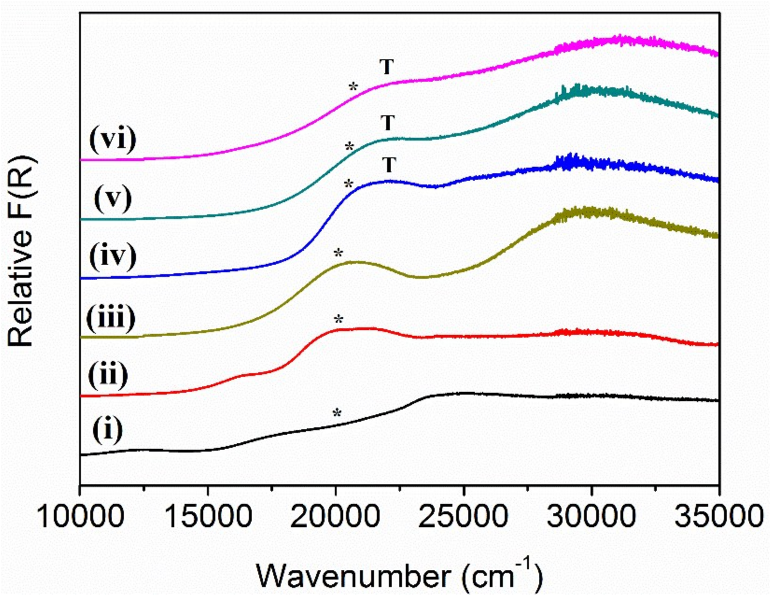

Figure S3: Solid State UV-Vis-NIR measurements of **(i) Co1 (ii) Co2 (iii) Co3 (iv) POPCo1 (vi) POPCo2 (v) POPCo3**. * denotes the shift in bands from the discrete complexes to the POPs, while the **T** denotes the band that appears from the TPA co-ligand.

Table S1:ICP-OES results for Co(III) salen polymers

POP	Suspected Co content (%)	Found (%)
**POPCo1**	8.38	6.31
**POPCo2**	8.45	6.65
**POPCo3**	8.13	5.83

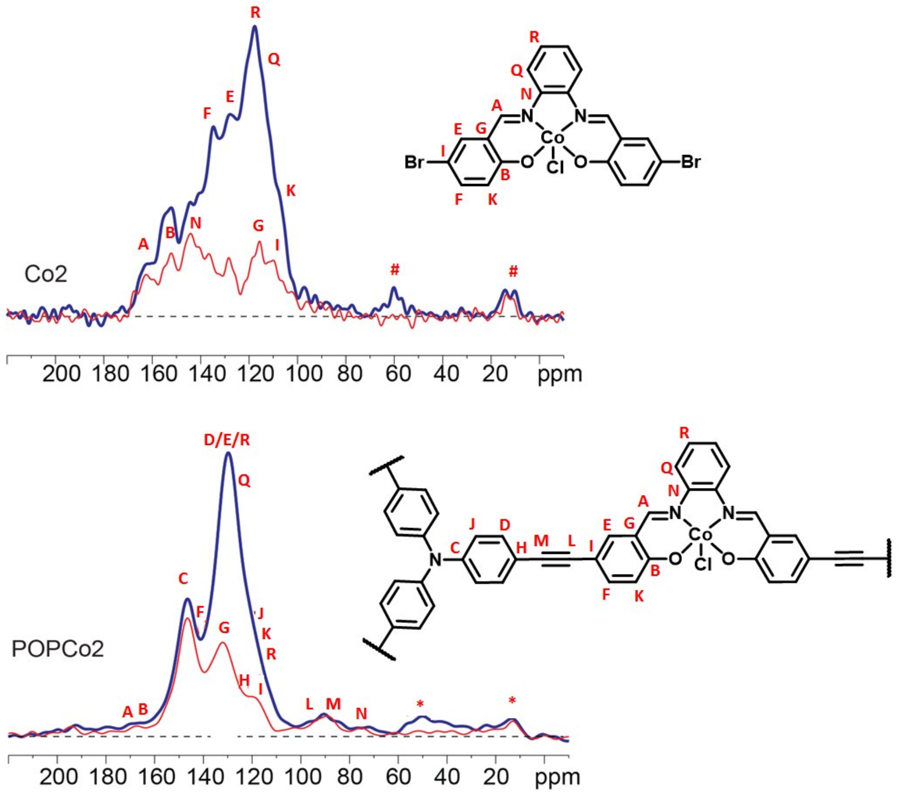

Figure S4: ^{13}C CPTOSS of **Co2** (above) and **POPCo2** (below). The full ^{13}C NMR spectra are plotted in blue, while the spectra in red are the non-protonated or methyl carbon species detected after 40 µs of dipolar dephasing. Residual ethanol is denoted with a #, while residual triethylamine is noted with a *

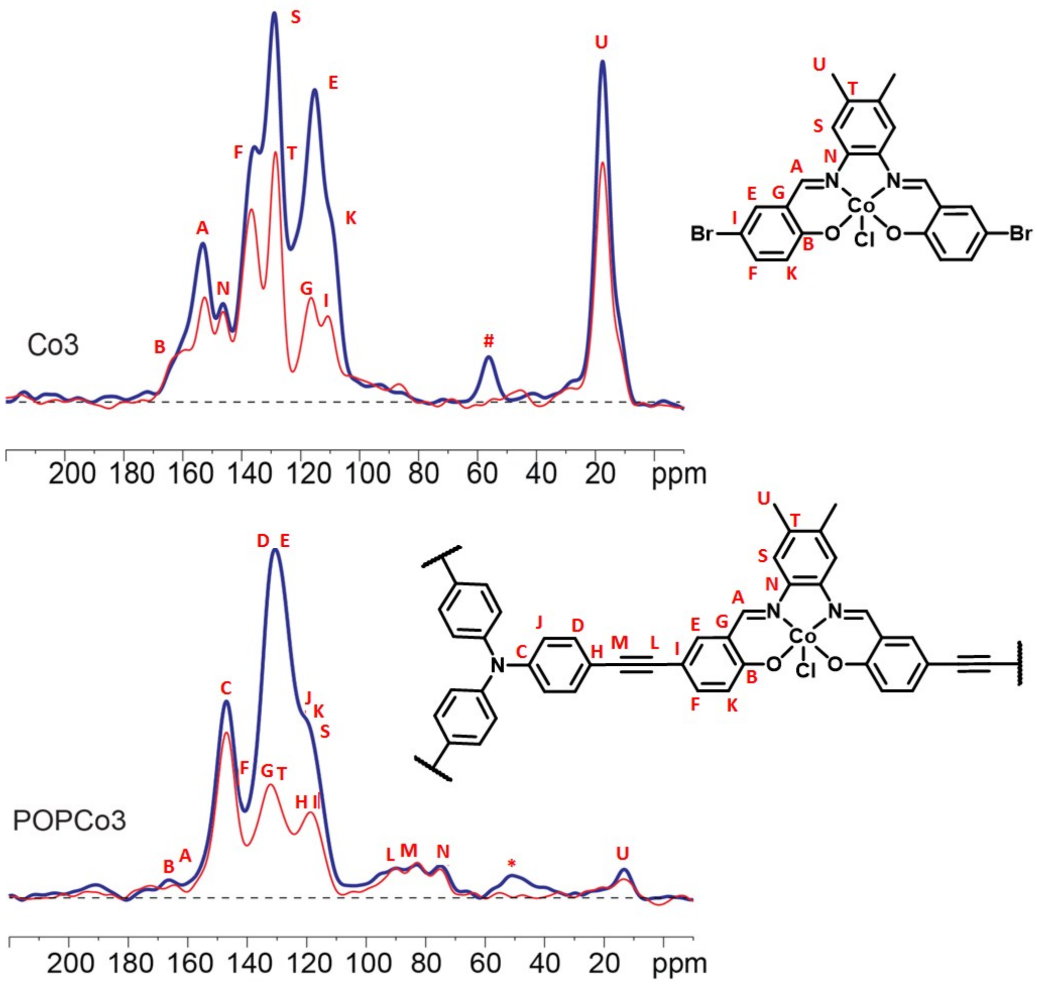

Figure S5: ^{13}C CPTOSS of **Co3** (above) and **POPCo3** (below) the aromatic salen POPs, as well as the POP made in the absence of salen metal complex. The full ^{13}C NMR spectra are plotted in blue, while the spectra in red are the non-protonated or methyl carbon species detected after 40 μs of dipolar dephasing. Residual ethanol is denoted with a #, while residual triethylamine is noted with a *

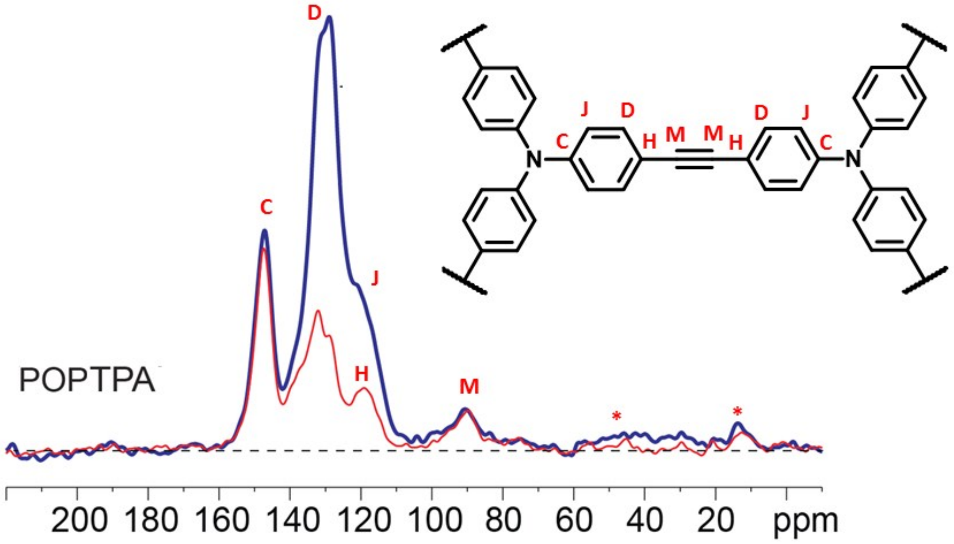

Figure S6: ^{13}C CPTOSS of **POPTPA**. The full ^{13}C NMR spectra are plotted in blue, while the spectra in red are the non-protonated or methyl carbon species detected after 40 μs of dipolar dephasing. Residual triethylamine is noted with a *

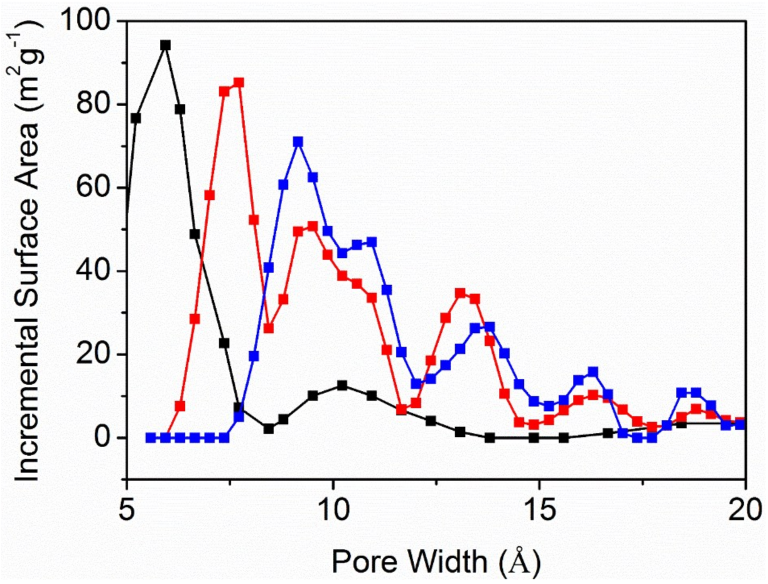

Figure S7: DFT pore size distributions for **POPCo1** (black), **POPCo2** (red) and **POPCo3** (blue).

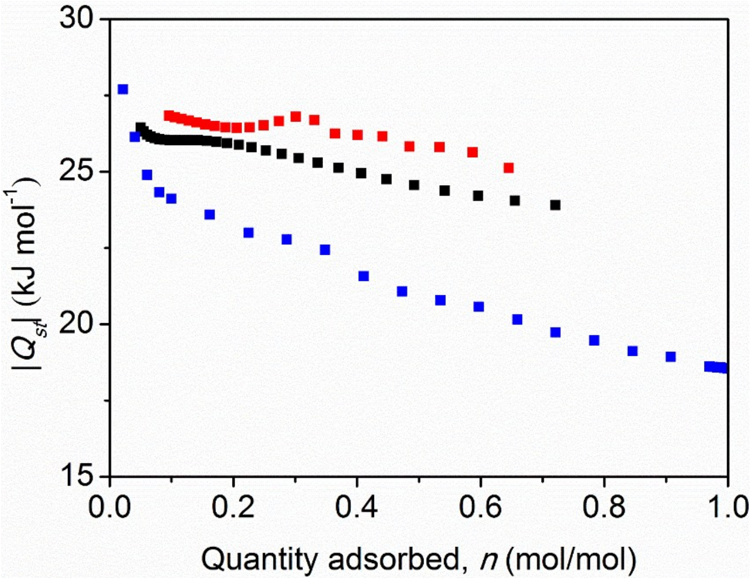

Figure S8: Isosteric heats of adsorption for **POPCo1** (black), **POPCo2** (red) and **POPCo3** (blue).

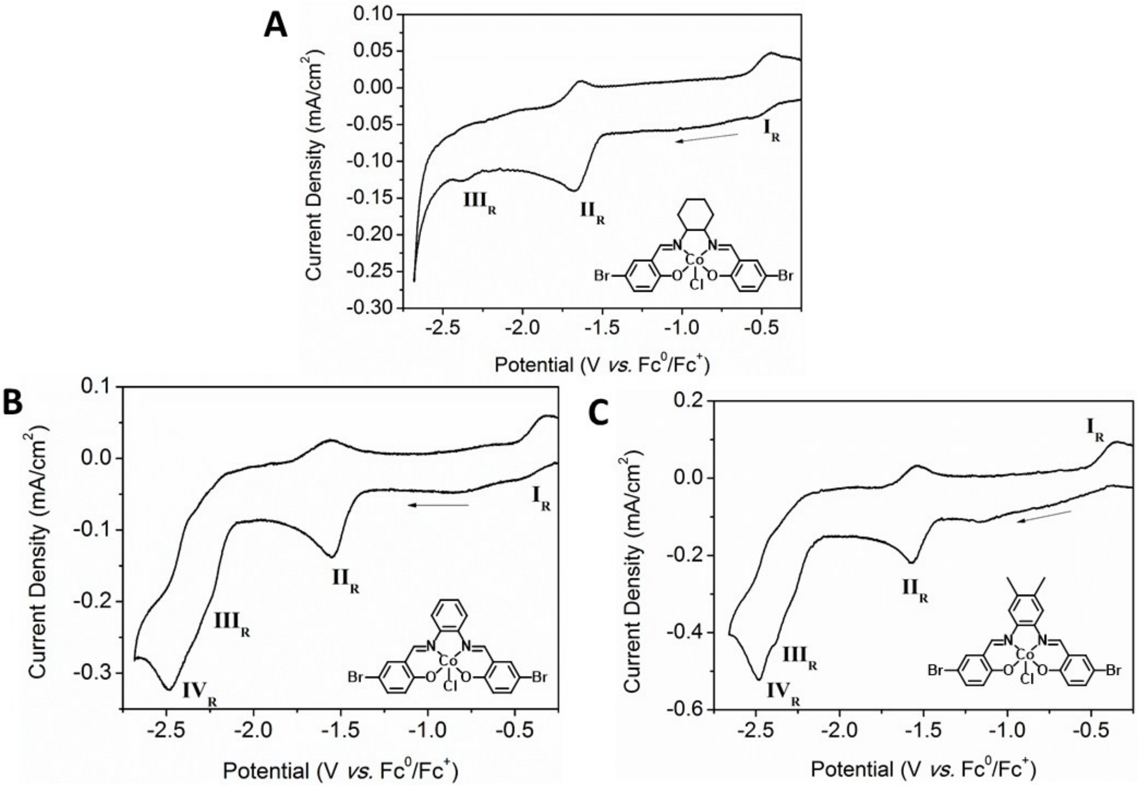

Figure S9: Solution state CV of **A Co1** (1 mM), **B Co2** (1 mM) and **C Co3** (1 mM). (0.1 M [(n-C_4H_9)$_4$N]PF_6/DMF as the supporting electrolyte under N_2, scan rate: 0.1 V s^{-1}, Fc (1 mM) was used as an internal standard).

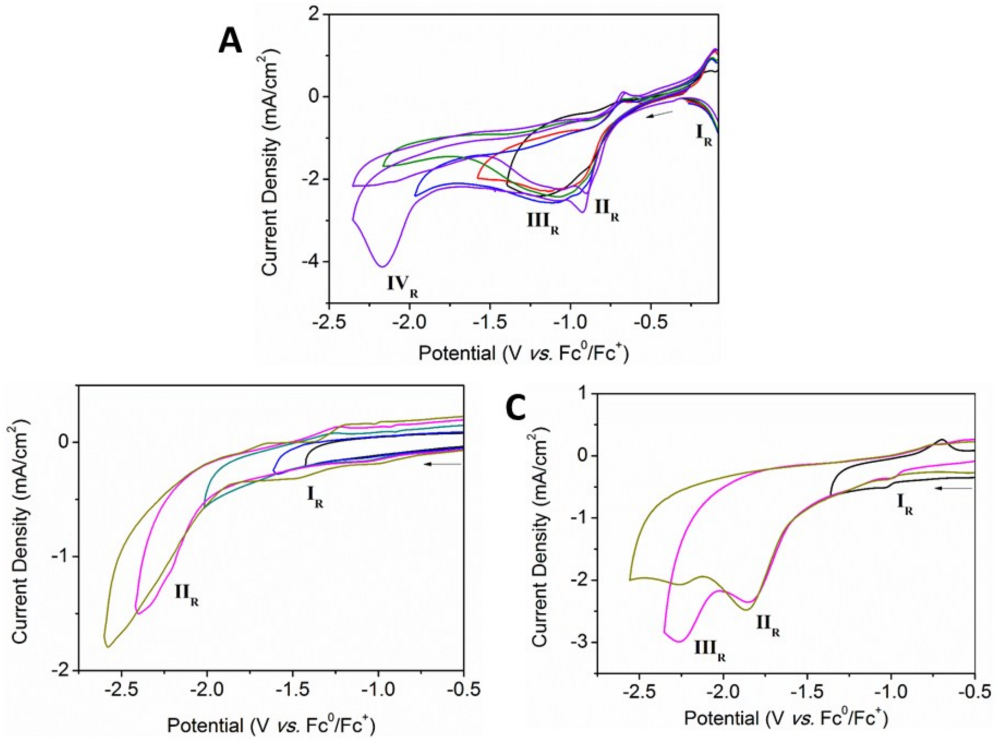

Figure S10: Solid state CV of **A POPCo1** upon progressively extending the potential window to E_{pc} = −1.39 (black), −1.58 (red), −1.96 (blue), −2.17 (green), −2.36 V vs. Fc^0/Fc^+ (purple) **B POPCo2** upon progressively extending the potential window to E_{pc} = −1.42 (black), −1.62 (blue), −2.01 (green), −2.41 (magenta) and −2.58 V vs. Fc^0/Fc^+ (yellow) and **C POPCo3** upon progressively extending the potential window to −1.36 (black), −2.35 (magenta) and −2.55 V vs. Fc^0/Fc^+ (yellow), **b)** under N_2 (black), CO_2 (red) and CO_2 with TFE (0.14 mmol-blue, 0.28 mmol-green) (0.1 M $LiBF_4$/MeCN as the supporting electrolyte, scan rate: 0.025 V s^{-1}, Fc (1 mM) was used as an internal standard).

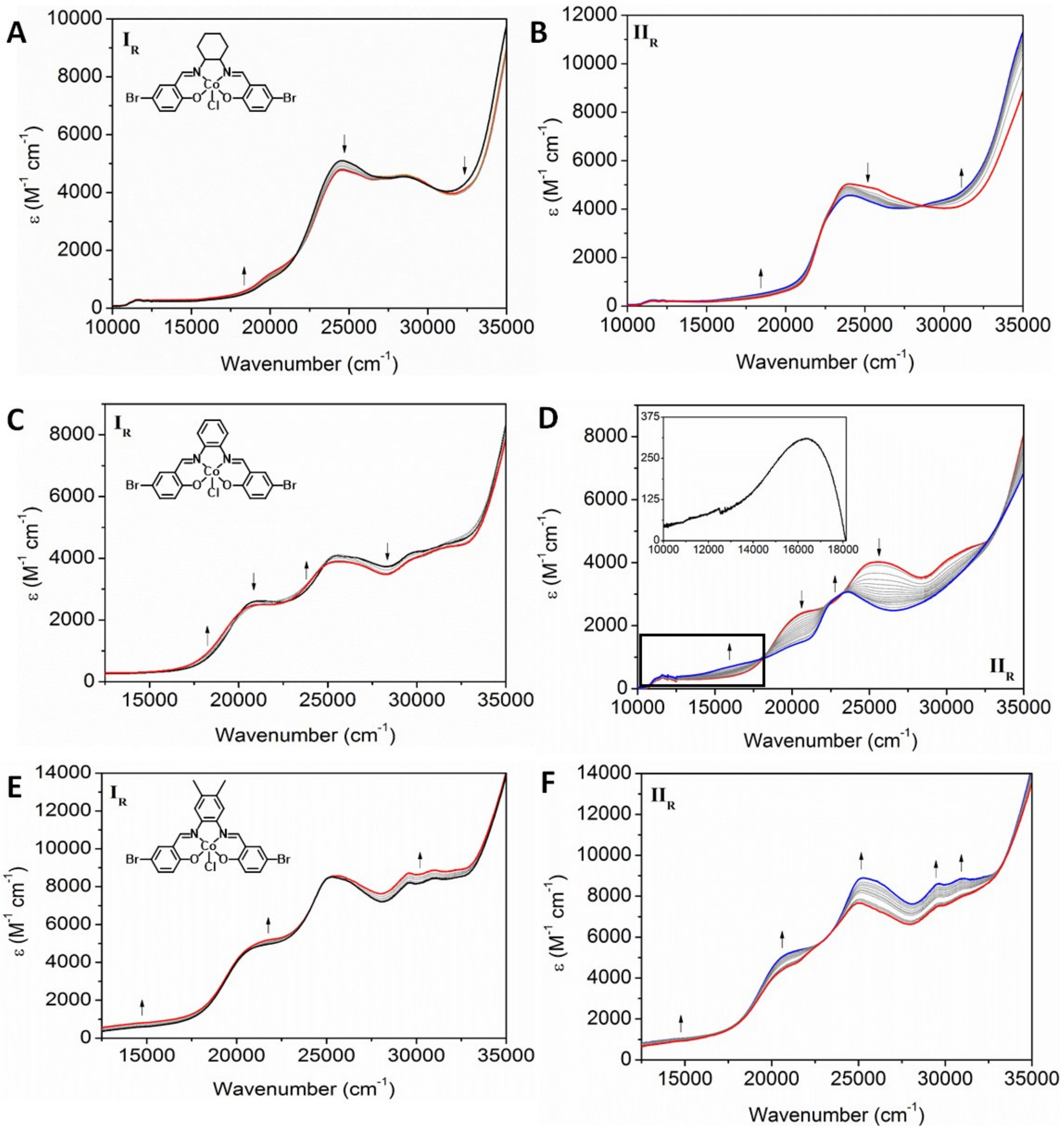

Figure S11: Solution state UV-Vis-NIR SEC of **A Co1** (0.61 mM) upon changing the potential from +0.1 to −0.2 V vs. Ag/Ag^+ and **B** −1.0 to −1.5 V vs. Ag/Ag^+ **C Co2 (**0.51 mM) upon changing the potential from 0.1 to −0.3 V vs. Ag/Ag^+ and **D** −1.0 to −1.5 V vs. Ag/Ag^+ **E Co3** (0.32 mM) upon changing the potential from +0.1 to −0.4 V vs. Ag/Ag^+ and **F** −0.8 to −1.4 V vs. Ag/Ag^+. 0.1 M $[(n\text{-}C_4H_9)_4N]PF_6$/DMF as the supporting electrolyte.

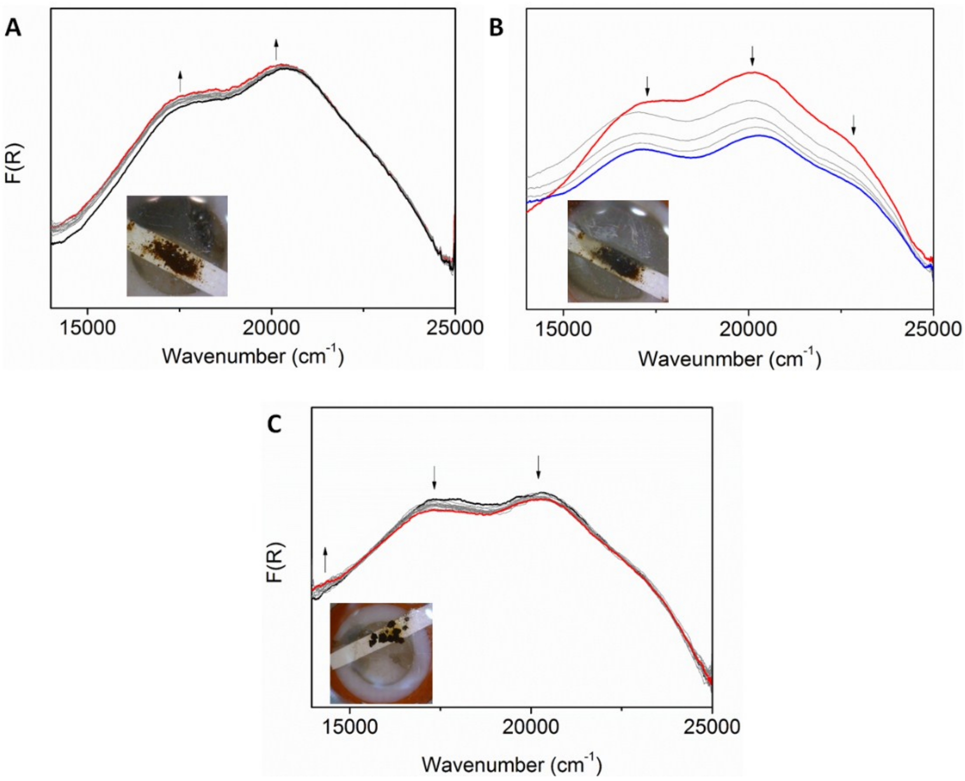

Figure S12: Solid state Vis-NIR SEC of **POPCo1** upon changing the potential from **A** +0.1 to −0.4 V vs. Ag/Ag$^+$, **B** −0.8 to −1.4 V vs. Ag/Ag$^+$ and **C** of **POPCo2** upon changing the potential from −0.9 to −1.3 V vs. Ag/Ag$^+$ (0.1 M $[(n\text{-}C_4H_9)_4N]PF_6$/MeCN as the supporting electrolyte. The insets show the polymers during the experiment.

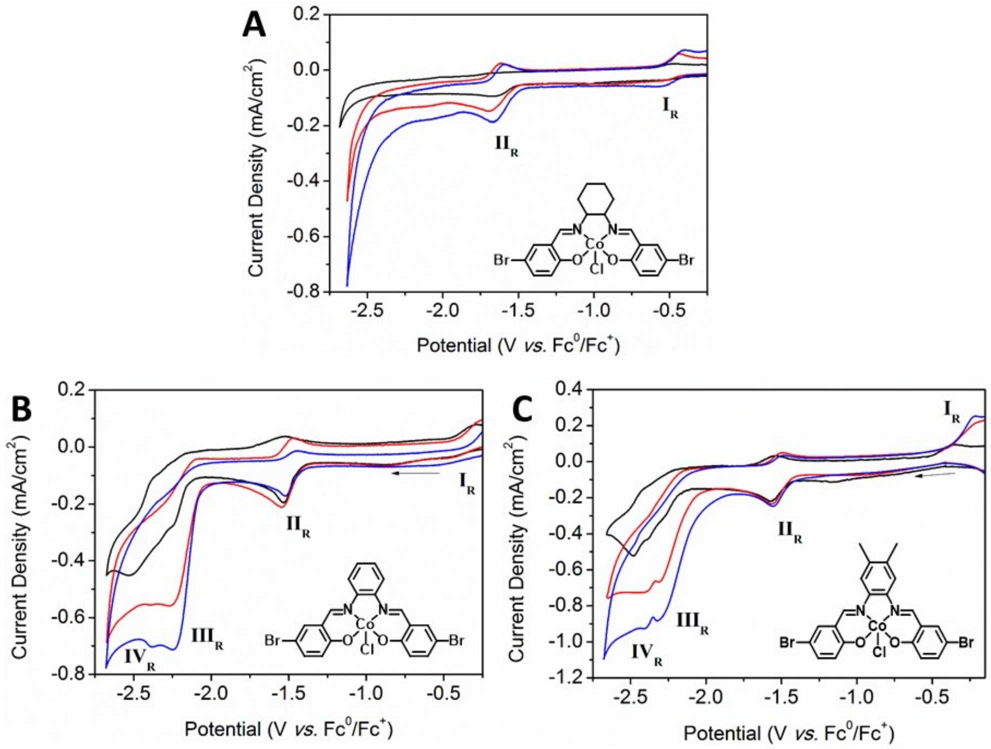

Figure S13: Solution state CV showing the electrochemical response of **A Co1** (1 mM) under saturation conditions of N_2 (black), CO_2 (red) and CO_2 with TFE (2.1 mmol) as a proton source (blue) **B Co2** (1 mM) under saturation conditions of N_2 (black), CO_2 (red) and CO_2 with TFE (6.3 mmol) as a proton source (blue) **C Co3** (1 mM) under saturation conditions of N_2 (black), CO_2 (red) and CO_2 with TFE (2.1 mmol) as a proton source (blue) (0.1 M $[(n\text{-}C_4H_9)_4N]PF_6$/DMF as the supporting electrolyte, scan rate: 0.1 V s^{-1}, Fc (1 mM) was used as an internal standard).

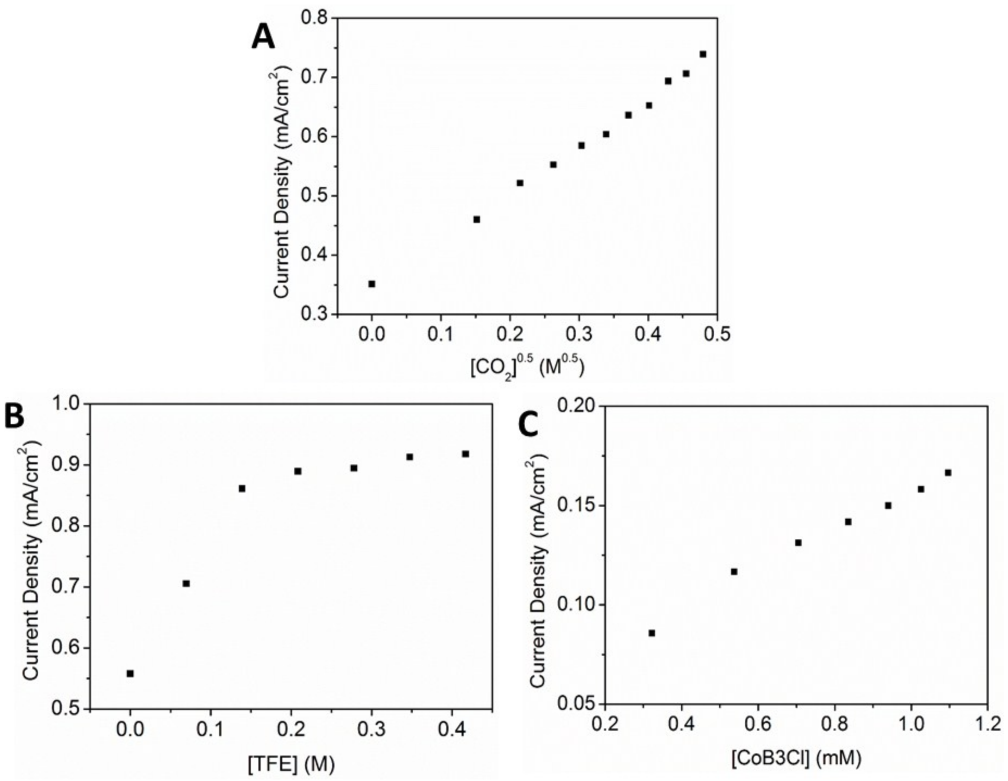

Figure S14: Plot of **A** current density (i_{cat}) vs. $[CO_2]^{0.5}$ for solution state CV of **Co2** (1 mM) under CO_2, demonstrating a first order kinetic relation in $[CO_2]$ **B** i_{cat} vs. [TFE] for solution state CV of **Co2** (1 mM) under CO_2 saturation, demonstrating a second order kinetics relation in [TFE] and **C** i_{cat} vs. [**Co2**] for solution state CV of **Co2** under CO_2 saturation, demonstrating a first order kinetic relation in [**Co2**].

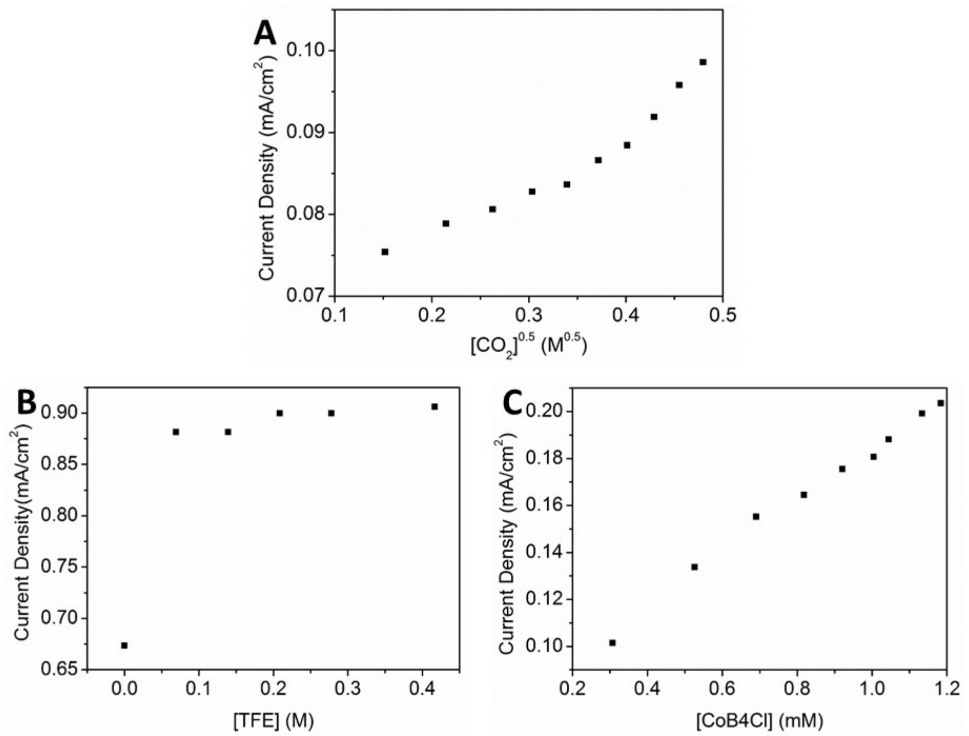

Figure S15: Plot of **A** current density (i_{cat}) vs. $[CO_2]^{0.5}$ for solution state CV of **Co3** (1 mM) under CO_2, demonstrating a first order kinetic relation in $[CO_2]$ **B** i_{cat} vs. [TFE] for solution state CV of **Co3** (1 mM) under CO_2 saturation, demonstrating a second order kinetics relation in [TFE] and **C** i_{cat} vs. [**Co2**] for solution state CV of **Co2** under CO_2 saturation, demonstrating a first order kinetic relation in [**Co3**].

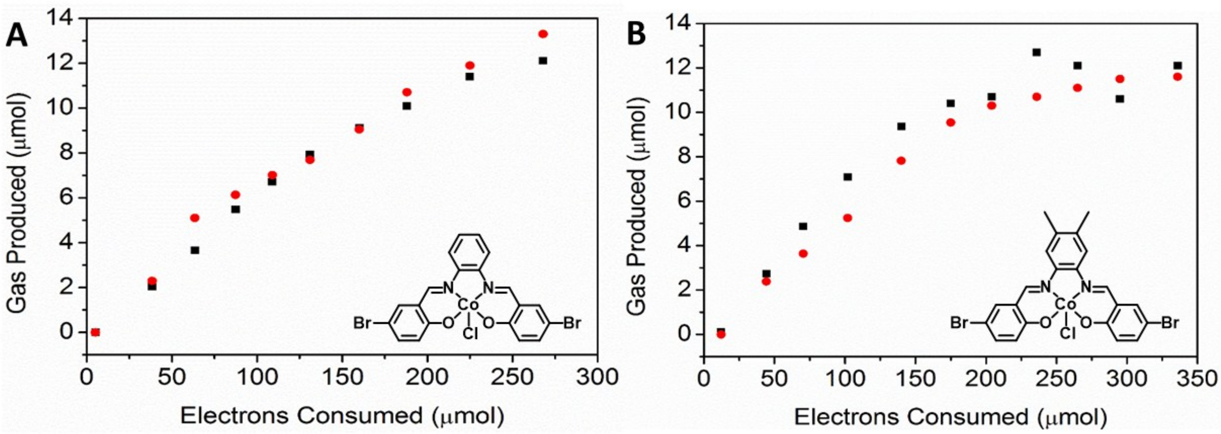

Figure S16: Production of H_2 (black) and CO (red) from CO_2 by **A Co2** (3.60 mM) with TFE (0.63 M) and **B Co3** (1.11 mM) and (0.14 M) during CPE (0.1 M [(n-$C_4H_9)_4N]PF_6$/DMF/MeCN(8:2) with TFE (0.63 M) as the supporting electrolyte under CO_2, $E_{pc} = -1.85$ V vs. Ag/Ag$^+$).

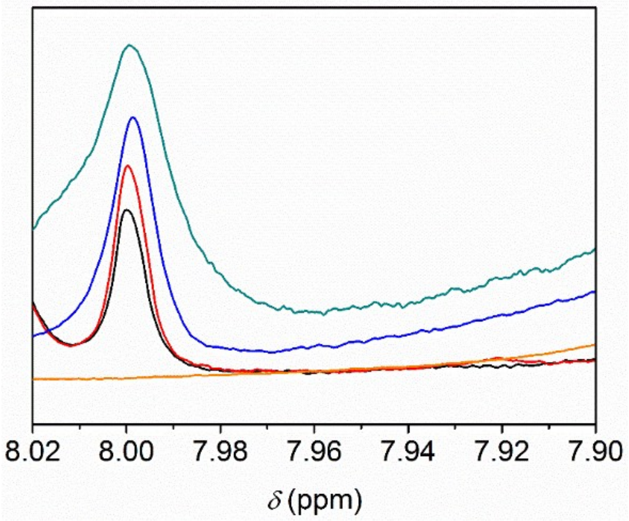

Figure S17: ^{1}H NMR of the **Co2** bulk electrolysis solution after work up from CPE in D_2O at 300 MHz under CO_2 saturation after 30 min (black), 60 min (red), 90 min (blue), 120 min (cyan) and in the absence of CO_2 after 120 min (orange). The peak at $\delta = 8.00$ ppm corresponds to the generation of formic acid. Spectra were referenced to D_2O. $E_{pc} = -1.85$ V vs. Ag/Ag$^+$.

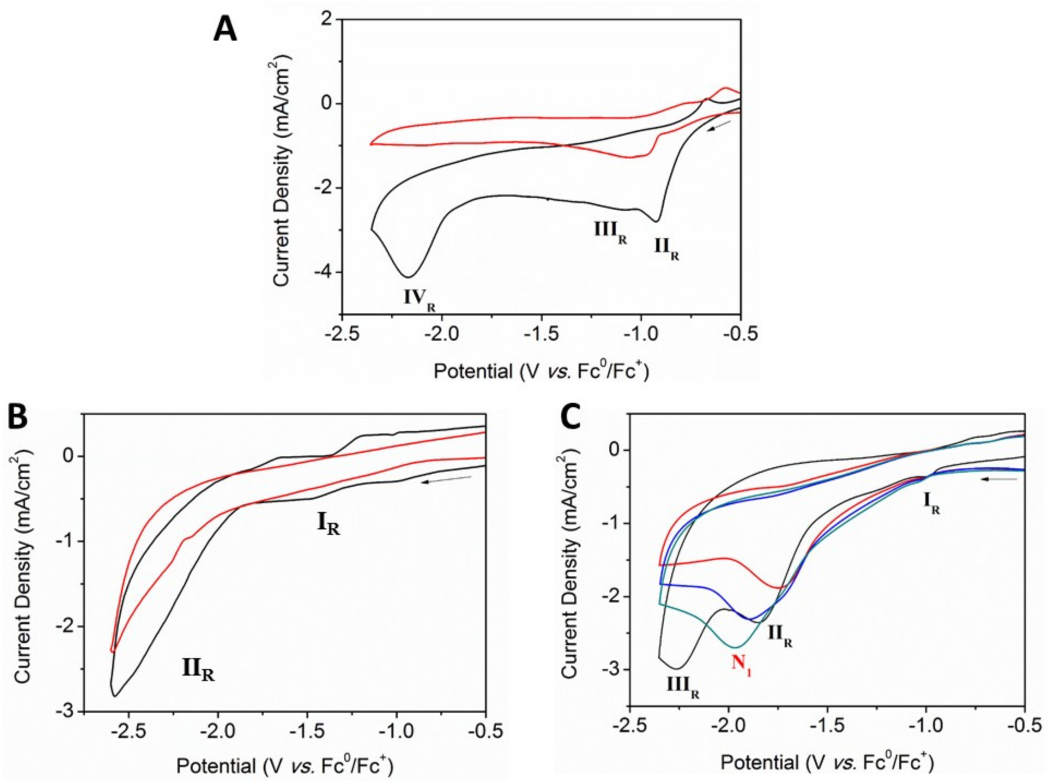

Figure S18: Solid state CV of **A POPCo1 B POPCo2** and **C POPCo3** under N_2 (black) and under CO_2 (red) with TFE (0.14 mmol-blue, 0.28 mmol-green) (0.1 M $LiBF_4$/MeCN as the supporting electrolyte, scan rate: 0.025 V s^{-1}, Fc (1 mM) was used as an internal standard). N_1 denotes the new

References

1. Hua, C.; Chan, B.; Rawal, A.; Tuna, F.; Collison, D.; Hook, J. M.; D'Alessandro, D. M. Redox tunable viologen-based porous organic polymers. J. Mater. Chem. C. **2016,** 4, 2535-2544 DOI: 10.1039/C6TC00132G.
2. Coulson, D. R.; Satek, L. C.; Grim, S. O. In Inorg. Synth.; John Wiley & Sons, Inc.: 2007; pp 121-124.
3. Szłyk, E.; Wojtczak, A.; Surdykowski, A.; Goździkiewicz, M. Five-coordinate zinc(II) complexes with optically active Schiff bases derived from (1R,2R)-(−)cyclohexanediamine: X-ray structure and CP MAS NMR characterization of [cyclohexylenebis(5-chlorosalicylideneiminato)zinc(II)pyridine] and [cyclohexylenebis(5-bromosalicylideneiminato)zinc(II)pyridine]. Inorg. Chim. Acta **2005,** 358, 467-475 DOI: http://dx.doi.org/10.1016/j.ica.2004.07.065.
4. Giannicchi, I.; Brissos, R.; Ramos, D.; Lapuente, J. d.; Lima, J. C.; Cort, A. D.; Rodríguez, L. Substituent Effects on the Biological Properties of Zn-Salophen Complexes. Inorg. Chem. **2013,** 52, 9245-9253 DOI: 10.1021/ic4004356.
5. Uh, H.; Badger, P. D.; Geib, S. J.; Petoud, S. Synthesis and Solid-State, Solution, and Luminescence Properties of Near-Infrared-Emitting Neodymium(3+) Complexes Formed with Ligands Derived from Salophen. Helv. Chim. Acta **2009,** 92, 2313-2329 DOI: 10.1002/hlca.200900162.
6. Smith, H. E.; Neergaard, J. R.; Burrows, E. P.; Chen, F.-M. Optically active amines. XVI. Exciton chirality method applied to the salicylidenimino chromophore. Salicylidenimino chirality rule. J. Am. Chem. Soc. **1974,** 96, 2908-2916 DOI: 10.1021/ja00816a041.
7. Latif, N.; Mishriky, N.; Assad, F. M. Carbonyl and thiocarbonyl compounds XX. Reaction of hydroxybenzaldehydes with o-phenylenediamine; newer aspects in benzimidazole synthesis. Recl. Trav. Chim. Pays-Bas **1983,** 102, 73-77 DOI: 10.1002/recl.19831020203.
